# DMP1-Cre expressing cells mediate the gain in bone mass and strength, but not the increase in bone remodeling, induced by ligands of the parathyroid hormone receptor

**DOI:** 10.1038/s41413-026-00555-z

**Published:** 2026-07-30

**Authors:** Nisreen Akel, Sasidhar Uppuganti, Jeff D. Thostenson, Wyatt House, Kaitlyn Hapke, Gretel G. Pellegrini, Joan Pizarro-Gómez, Shenyang Li, Meloney Cregor, Silvia Marino, Jeffry S. Nyman, Teresita Bellido

**Affiliations:** 1 Department of Physiology and Cell Biology, University of Arkansas for Medical Sciences, Little Rock, AR USA; 2 Central Arkansas Veterans Healthcare System, John L. McClellan, Little Rock, AR USA; 3 Department of Orthopedic Surgery, Vanderbilt University Medical Center, Nashville, TN USA; 4 Department of Biostatistics, University of Arkansas for Medical Sciences, Little Rock, AR USA; 5 Department of Veterans Affairs, Tennessee Valley Healthcare System, Nashville, TN USA; 6 Winthrop P. Rockefeller Cancer Institute, University of Arkansas for Medical Sciences, Little Rock, AR USA

**Keywords:** Bone, Diabetes complications, Diabetes complications

## Abstract

Signaling downstream of the receptor of parathyroid hormone (PTH1R) exerts two major skeletal effects: increases bone remodeling and, when stimulated intermittently, induces bone anabolism. Osteocytes express the PTH1R and are critical for the action of teriparatide/parathyroid hormone 1–34 (PTH). However, it is unknown whether they also mediate the effects of abaloparatide (a 34 amino acid synthetic analog of human parathyroid hormone-related protein, ABL), and whether actions on osteocytes are required for the increase in remodeling and/or the bone gain induced by PTH/ABL in diabetes. We addressed these questions by treating with PTH or ABL control or diabetic (DM) mice lacking the PTH1R in DMP1-Cre expressing cells that targets all osteocytes (cKO). Both PTH and ABL increased bone mass and improved or corrected cortical and trabecular bone microarchitecture only in fl/fl littermates but not in cKO, control or DM mice. Further, PTH/ABL increased bone strength and microindentation resistance only in control or DM fl/fl mice. In contrast, PTH/ABL increased serum P1NP and bone formation on cancellous, periosteal and endocortical surfaces, in control or DM mice of both genotypes. Moreover, serum CTX and osteoclast surface were increased by PTH/ABL in fl/fl and cKO, control or DM mice. Thus, actions on DMP1-Cre expressing cells are required for bone gain, microarchitecture restoration, strength and resistance to fracture, exerted by PTH and ABL under physiological and DM conditions, but not for the increase in bone remodeling. These findings demonstrate the dissociation of bone gain from bone remodeling and reveal that actions on DMP1-Cre expressing cells drive the gain in bone mass and strength induced by PTH and ABL.

## Introduction

Diabetes Mellitus (DM) is a worldwide epidemic, with an expected prevalence reaching > 12% of 20–79-year-old individuals (~800 million) by 2045.^1^ DM affects multiple tissues and organs, being the musculoskeletal system highly impacted. Increased bone fracture risk is a major public health complication of this disease, as diabetic patients display augmented morbidity and mortality after a bone fracture, compared to normoglycemic individuals.^2,3^

Extensive research of the last few years demonstrates that DM markedly alters the process of bone remodeling by inducing an acute increase in bone resorption that subsides in chronic disease stages, concomitant to a rapid and sustained decrease in bone formation that remains low during the entire course of the disease.^4^ Thus, a feature of diabetic bone disease is deteriorated microarchitecture that cannot be rebuilt, and which might or might not be accompanied by changes in bone mineral density (BMD). Bone antiresorptive agents inhibit resorption and effectively stop the (further) bone loss; yet antiresorptives do not increase bone formation nor restore damaged architecture. Therefore, better therapeutic approaches to restore bone strength in DM are needed.

Post hoc analysis of large clinical osteoporosis trials showed that PTH, ABL, as well as the dual anabolic/antiresorptive agent romosozumab, increased BMD and trabecular bone score and reduced non-vertebral fracture incidence similarly in diabetic and non-diabetic patients,^5–8^ suggesting that maneuvers that induce bone anabolism are suitable therapeutic tools for DM-induced bone disease. Consistently, recent pre-clinical studies revealed that bone anabolism driven by activation of PTH receptor (PTH1R) signaling with PTH or ABL, as well as with romosozumab, corrected the features exhibited by diabetic bone at the tissue, cell and transcriptome levels.^9^ Both PTH and ABL increased BMD, corrected the deteriorated bone architecture and cortical porosity, and restored the mechanical properties of bone. The effect of the agents is due to a marked increase in bone formation and resorption with a positive balance leading to bone gain and to a reversal of the changes in the bone/bone marrow transcriptome induced by DM. However, ABL was more potent than PTH in increasing bone remodeling, activated many more genes and pathways compared to PTH, and increased bone toughness whereas PTH was not as effective. The mechanism(s) for the distinct potency of these two ligands is not known, and the different potency could be explained by their action on different bone/bone marrow target cells.

PTH1R is expressed in several cells of the osteoblastic lineage as well as in cells of the bone marrow microenvironment; and PTH has been shown to regulate the function of all these cell types. Among them, osteocytes are critical target cells of PTH action on bone. Activation of the PTH1R expressed in osteocytes is sufficient to stimulate bone remodeling,^10^ whereas its deletion using the DMP1-8kb-Cre deletor (DMP1-Cre) impairs bone anabolism induced by PTH as well as the anabolic response to mechanical stimulation.^11^ In the current study, we examined whether expression of the PTH1R in DMP1-Cre expressing cells is also required for the skeletal effects of ABL and whether it mediates the protective actions of both ligands of the PTH1R in the frame of DM. We addressed these questions using targeted deletion of the PTH1R from osteocytes with the DMP1-Cre deletor, which targets all osteocytes and mature osteoblasts. We found that DMP1-Cre expressing cells are major target cells responsible for the protective actions of both PTH1R ligands in DM, as mice lacking the PTH1R in DMP1-Cre expressing cells (cKO) did not exhibit increases in bone mass, microarchitecture restoration, or the improvement of several mechanical and material properties determinant of bone strength. Remarkably, however, expression of PTH1R in DMP1-Cre expressing cells is dispensable for the effect of PTH and ABL on enhancing the bone remodeling rate, as bone formation and resorption were increased by the ligands even in cKO mice. These findings demonstrate that the expression of the PTH1R in cells expressing DMP1-Cre is required for bone gain, microarchitecture repair, and bone strength induced by PTH and ABL, whereas it is dispensable for their effects on bone remodeling, under physiological and DM conditions. We conclude that deleting the PTH1R from DMP1-Cre expressing cells leads to dissociation of bone gain from increased bone remodeling induced by PTH/ABL, strongly suggesting that osteocytes mediate the gain in bone mass and strength induced by ligands of the parathyroid hormone receptor.

## Results

### Mice lacking PTH1R in DMP1-Cre–expressing cells developed diabetes similarly to fl/fl littermates and PTH/ABL had no effect on blood glucose or body weight

**Fig. 1:**
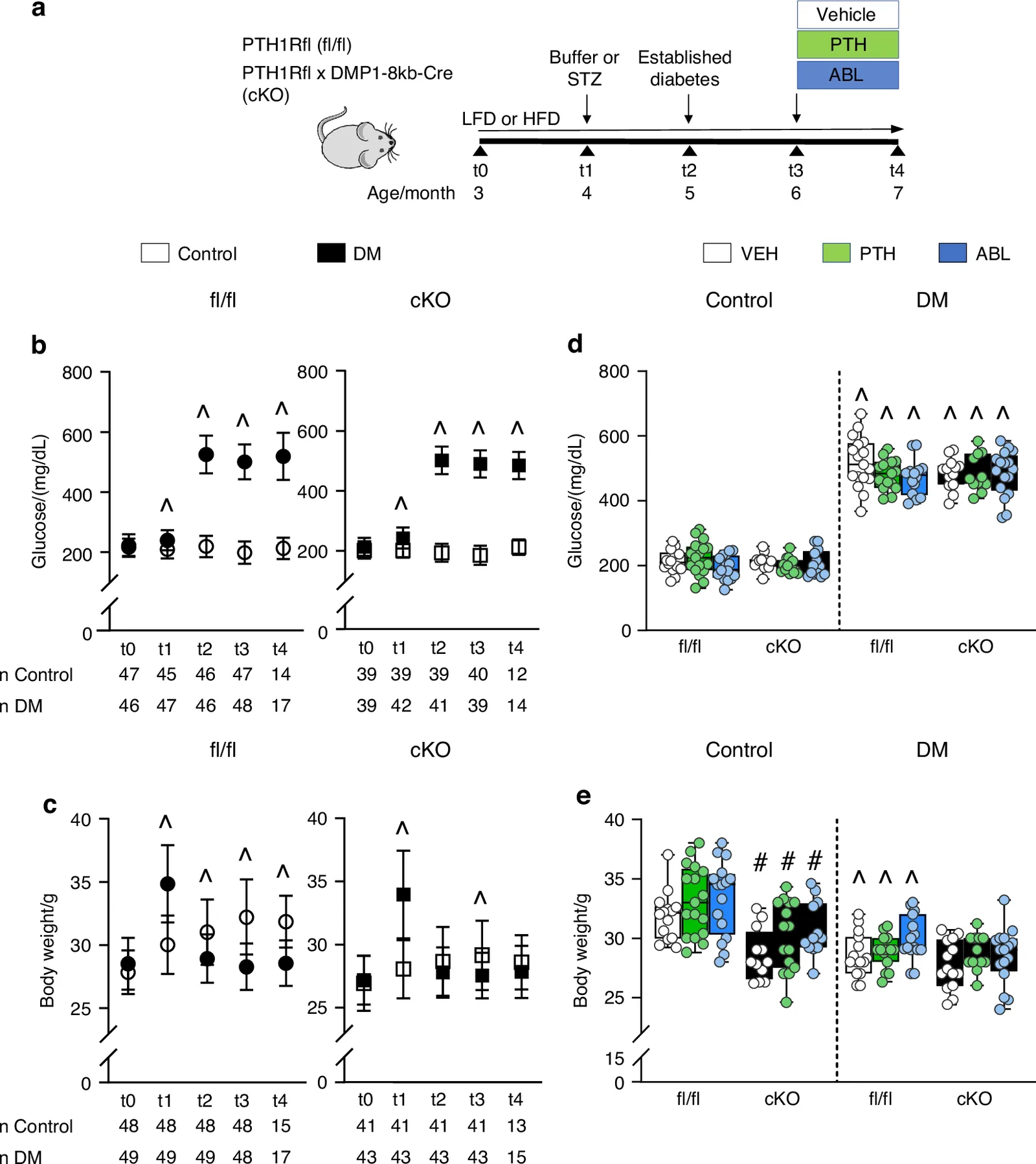
Mice lacking PTH1R in DMP1–Cre-expressing cells (cKO) developed diabetic bone disease similarly to control littermates (fl/fl) and treatment with PTH/ABL did not alter the diabetic condition. **a** Study design depicting the preclinical diabetes (DM) model. For details, see Methods. **b**, **d** Blood glucose concentration. **c**, **e** Body weight. For **b** and **c**, *n* = 39–49/group at t0, t1 and t2; *n* = 39–48/group at t3; and *n* = 12–17 at t4 (vehicle only). For **d** and **e**, *n* = 11–17 mice/group. ^*P* < 0.05 versus control mice by Repeated Measures models (**b**, **c**) and by three-way ANOVA with factors of DM, genotype, and treatment (**d**, **e**). #*P* < 0.05 versus respective fl/fl mice (**e**). Each dot represents a mouse

DM was induced by a combination of high-fat diet (HFD) and streptozotocin (STZ) in skeletally mature male conditionally knockout mice lacking the PTH1R in DMP1-Cre expressing cells (cKO) and control littermates (fl/fl) (Fig. 1a). Longitudinal BMD measurements revealed that cKO mice maintained in a low fat diet (control) exhibited a modest but significant increase in total and spinal BMD vs fl/fl littermate mice, as previously reported^11^ (Fig. S1a and 1c). The difference in BMD between genotypes was lost with the progression of DM. Overt hyperglycemia was observed in both genotypes at t2, with blood glucose > 250 mg/dL compared to non-diabetic control mice, which persisted throughout the study (Fig. 1b, Fig. S1d). cKO mice exhibit slightly lower body weight (Fig. S1e). Regardless, body weight increased similarly in fl/fl and cKO mice fed HFD for 1 month (t1) compared to LFD-fed control mice (Fig. 1c and Fig. S1e), due to a gain in fat mass (Fig. S2a). Progressively, body weight, fat mass and lean body mass decreased in DM mice from both genotypes, although the decrease in body weight and fat mass was less pronounced in the cKO mice (Fig. 1c and Fig. S2a and 2c). Administration of PTH or ABL had no effect on blood glucose levels, body weight, or fat and lean mass (Fig. 1d–e and Fig. S2b and 2d). Thus, mice from both genotypes developed DM, which was not affected by the bone anabolic agents. Diabetic mice of both genotypes exhibited decreased scapular fat, but no detectable changes were detected in the inguinal or gonadal fat induced by DM (Fig. S2e–g). The anabolic treatments had no effect on fat accumulation in any of these tissues.

### The expression of the PTH1R in DMP1-Cre expressing cells is required for the full increase in bone mineral density and improved bone architecture induced by PTH and ABL under physiological and diabetic conditions

**Fig. 2:**
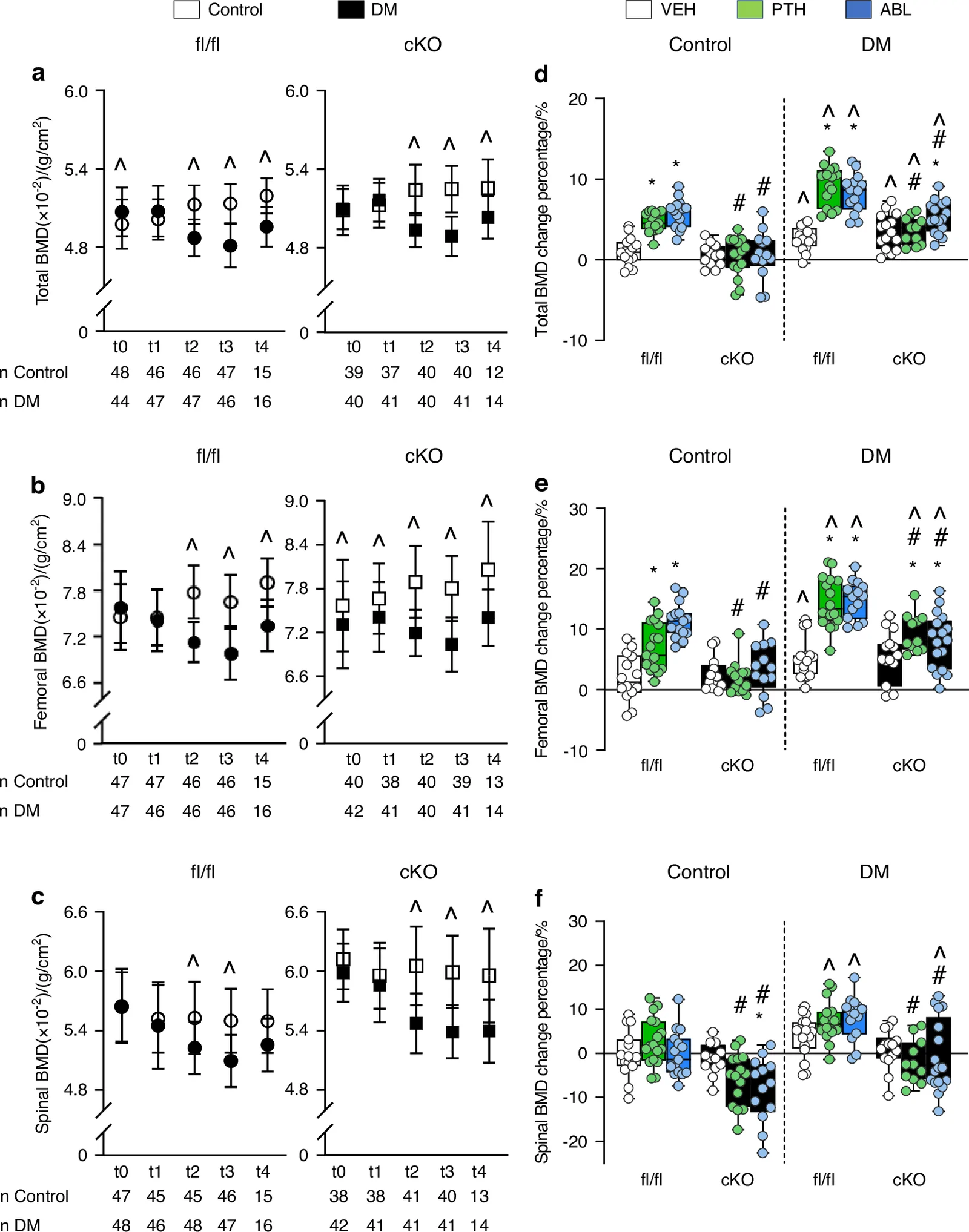
The expression of the PTH1R in DMP1-Cre cells is required for the full effect of PTH/ABL on bone mineral density (BMD). Longitudinal analysis of (**a**) total, (**b**) femoral, and (**c**) spinal BMD of cKO or fl/fl littermate control or DM mice. For **a–c**, *n* = 38–48/group at t0; *n* = 37–47/group at t1; *n* = 40–48/group at t2; *n* = 39–47/group at t3; and *n* = 12–16/group at t4 (vehicle only); and ^*P* < 0.05 versus control mice by Repeated Measures models. For (**d**–**f**), percent change BMD for each mouse was calculated following the formula (t4-t3) / t3 x 100; *n* = 11–17 mice/group. *P < 0.05 treatment versus respective vehicle treated mice; ^*P* < 0.05 DM versus control mice, and #*P* < 0.05 cKO versus fl/fl mice, by three-way ANOVA with factors of DM, genotype, and treatment. Each dot represents a mouse

**Fig. 3:**
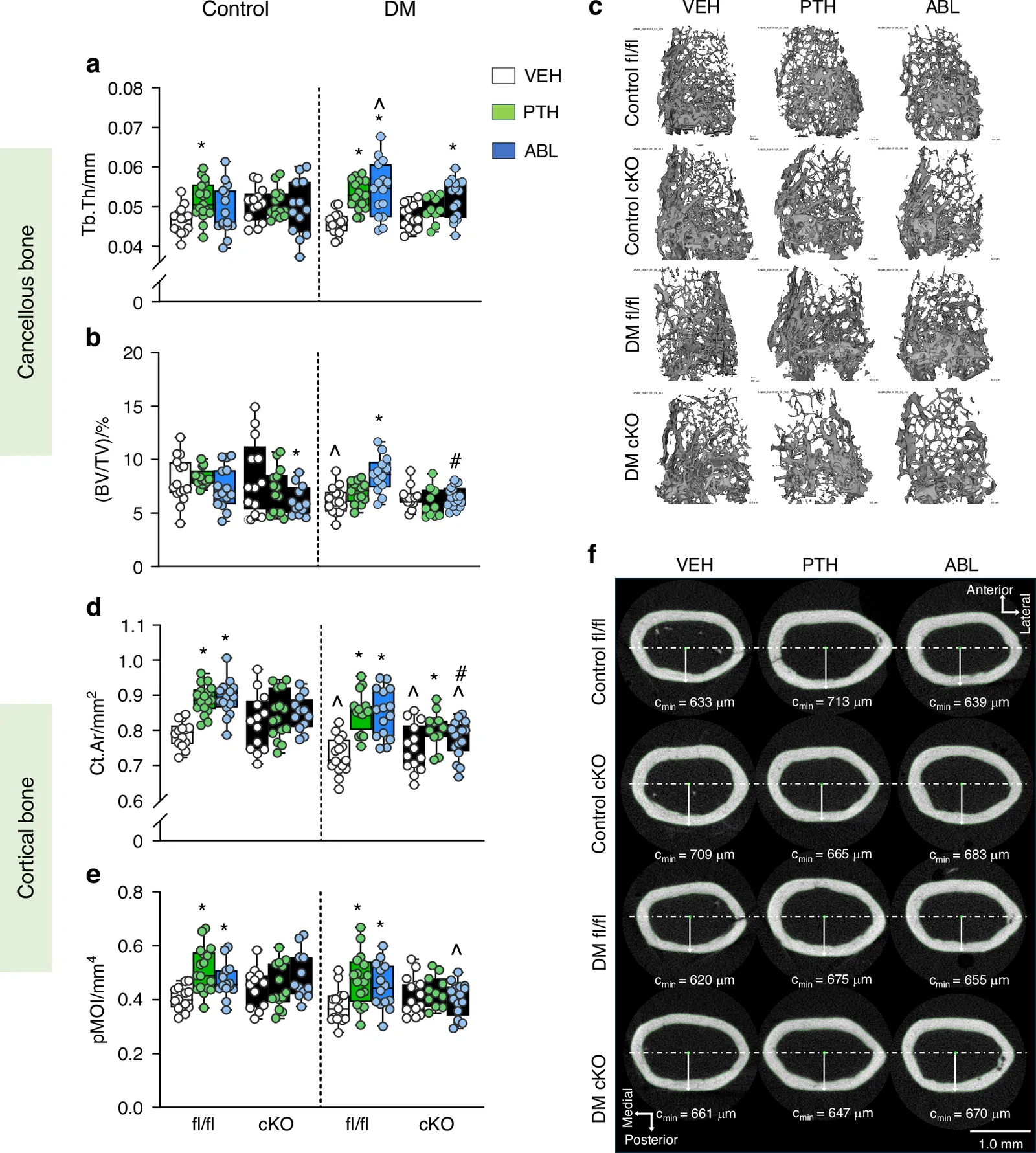
PTH and ABL corrected DM-induced deterioration in bone architecture in fl/fl mice but not in cKO mice. Micro-CT analysis of femoral cancellous (**a–c**) and femoral cortical (**d–f**) bone of fl/fl and cKO, control and DM mice; *n* = 10–17 mice/group. *P < 0.05 treatment versus respective vehicle treated mice; ^*P* < 0.05 DM versus control mice, and #*P* < 0.05 cKO versus fl/fl mice, by three-way ANOVA with factors of DM, genotype, and treatment. Each dot represents a mouse. **c** and **f** show representative micro-CT images

DM caused similar reduction in BMD in both cKO and fl/fl mice (Fig. 2a–c). The decreased total, femoral, and spinal BMD in DM mice was detected at t2 and BMD remained lower compared to control throughout the study. Based on earlier studies showing that ABL is more potent than PTH,^9,12^ we used doses of the ligands known to produce similar optimal bone effects (100 and 47.5 µg/kg/d, for PTH and ABL, respectively). Four weeks of treatment with PTH or ABL increased total and femoral BMD in control and restored the bone lost with DM in fl/fl mice but not control cKO mice and treatments were not fully effective in DM cKO mice (Fig. 2d–e). No major effects of PTH/ABL were seen on spinal BMD (Fig. 2f). PTH and ABL also improved bone microarchitecture in fl/fl mice but not in cKO mice under physiological or diabetic conditions (Fig. 3). PTH increased trabecular thickness (Tb.Th) in control fl/fl mice and both ABL and PTH similarly increased Tb.Th in DM fl/fl mice (Fig. 3a and Table 1**)**. Moreover, PTH/ABL did not increase Tb.Th in control cKO mice and only ABL was effective in diabetic cKO mice (Fig. 3a and c). DM induced a decrease in BV/TV in fl/fl mice that was restored by ABL, whereas PTH was not effective (Fig. 3b and c, Fig. S3a, and Table 1). Moreover, cancellous BV/TV of the lumbar vertebra 6 (L6) was increased by PTH/ABL significantly less in cKO DM versus fl/fl DM mice (Fig. S4a and Table 2). Consistent with the results in spinal BMD shown in Fig. S1c, Tb.Th was higher in control cKO mice compared to fl/fl mice, which was decreased by DM to fl/fl levels (Fig. S4b and Table 2). Further, PTH/ABL increased Tb.Th in control fl/fl mice but not in control cKO mice. Moreover, PTH/ABL increased Tb.Th in DM cKO mice significantly less than in DM fl/fl mice (Fig. S4b and Table 2).

**Table 1:** Femoral bone structural indices quantified by micro-CT and caliper of control and DM fl/fl and cKO mice treated with PTH or ABL

Femoral bone structural indices		Control			DM		
		Veh	PTH_100_	ABL_47.5_	Veh	PTH_100_	ABL_47.5_
Distal metaphysis							
(BV/TV)/% (*n* = 10–17)	fl/fl	8.05 ± 2.14	8.34 ± 0.84	7.40 ± 1.95	6.17 ± 1.28^	6.92 ± 1.04	8.64 ± 1.69*
	cKO	8.39 ± 3.49	7.10 ± 2.10	6.15 ± 1.39*	6.44 ± 1.02	5.95 ± 1.38	6.49 ± 0.95^#^
Tb.Th/mm (*n* = 10–17)	fl/fl	0.047 ± 0.003	0.052 ± 0.005*	0.49 ± 0.006	0.046 ± 0.003	0.053 ± 0.004*	0.054 ± 0.007^*
	cKO	0.051 ± 0.004	0.051 ± 0.004	0.050 ± 0.007	0.047 ± 0.003	0.049 ± 0.003	0.052 ± 0.005*
Tb.N/mm^−1^ (*n* = 11–16)	fl/fl	3.64 ± 0.30	3.28 ± 0.35*	3.20 ± 0.41*	3.34 ± 0.33^	3.0 ± 0.29*^	3.12 ± 0.50
	cKO	3.39 ± 0.29	2.96 ± 0.41*^#^	2.63 ± 0.39*^#^	3.30 ± 0.18	2.90 ± 0.23*	2.91 ± 0.27*
Conn.D/mm^−3^ (*n* = 11–17)	fl/fl	59.6 ± 25.8	98.43 ± 38.6*	110.8 ± 58*	46.1 ± 20.1	65.9 ± 18^	75.1 ± 29.4^*
	cKO	52.1 ± 24.6	70.4 ± 24.1	70.0 ± 19^#^	43.9 ± 12.2	57.1 ± 21.6	60.2 ± 20.4
SMI (*n* = 10–17)	fl/fl	2.75 ± 0.38	2.41 ± 0.35	2.56 ± 0.39	2.84 ± 0.30	2.72 ± 0.31	2.43 ± 0.51
	cKO	2.53 ± 0.56	2.50 ± 0.49	2.38 ± 0.49	2.83 ± 0.25	2.78 ± 0.30	2.7 ± 0.15
Tb.BMD/(mgHA/cm^3^) (*n* = 10–16)	fl/fl	83.9 ± 19.0	85.0 ± 15.7	76.3 ± 20.0	60.9 ± 14.1^	75.4 ± 18.9	83.2 ± 20.2*
	cKO	85.3 ± 35.9	71.1 ± 19.3	65.1 ± 13.9	63.3 ± 12.7^	59.4 ± 12.5	64.2 ± 7.3^#^
Tb.Sp/mm (*n* = 11–16)	fl/fl	0.27 ± 0.03	0.31 ± 0.04*	0.31 ± 0.03*	0.29 ± 0.03	0.33 ± 0.04*	0.33 ± 0.06*
	cKO	0.30 ± 0.03^#^	0.33 ± 0.05*	0.38 ± 0.06*^#^	0.30 ± 0.02	0.35 ± 0.03*	0.34 ± 0.04*^
Mid-diaphysis							
Ct.Th/mm (*n* = 11–16)	fl/fl	0.166 ± 0.01	0.173 ± 0.01*	0.180 ± 0.01*	0.152 ± 0.01^	0.168 ± 0.01*	0.166 ± 0.01^*
	cKO	0.162 ± 0.01	0.167 ± 0.01	0.166 ± 0.01^#^	0.153 ± 0.01^	0.164 ± 0.01*	0.158 ± 0.01^^#^
Ct.Ar/mm^2^ (*n* = 11–17)	fl/fl	0.79 ± 0.04	0.89 ± 0.04*	0.89 ± 0.05*	0.73 ± 0.05^	0.85 ± 0.06*	0.85 ± 0.07*
	cKO	0.82 ± 0.08	0.84 ± 0.07	0.85 ± 0.05	0.75 ± 0.07^	0.80 ± 0.05*	0.77 ± 0.05^^#^
Tt.Ar/mm^2^ (*n* = 10–17)	fl/fl	1.90 ± 0.13	2.12 ± 0.23*	1.97 ± 0.12	1.91 ± 0.23	2.06 ± 0.27*	1.99 ± 0.23
	cKO	2.03 ± 0.21	2.01 ± 0.22	2.12 ± 0.25	1.97 ± 0.16	1.89 ± 0.15^#^	1.92 ± 0.16^
MA/mm^2^ (*n* = 10–17)	fl/fl	1.11 ± 0.11	1.23 ± 0.21	1.10 ± 0.11	1.14 ± 0.14	1.21 ± 0.24	1.15 ± 0.17
	cKO	1.25 ± 0.19	1.17 ± 0.16	1.31 ± 0.25^#^	1.21 ± 0.12	1.14 ± 0.12	1.16 ± 0.11
(BA/TA)/% (*n* = 11–17)	fl/fl	41.40 ± 2.57	42.22 ± 3.56	45.19 ± 1.66*	39.49 ± 2.37	41.77 ± 3.83	41.65 ± 2.61^
	cKO	40.51 ± 1.62	41.83 ± 2.44	40.02 ± 3.33^#^	38.61 ± 2.82	40.84 ± 3.29	39.85 ± 1.61
Ct.TMD/(mgHA/cm^3^) (*n* = 11–17)	fl/fl	1247 ± 20	1237 ± 16	1236 ± 11	1240 ± 20	1238 ± 14	1232 ± 17
	cKO	1226 ± 16^#^	1232 ± 14	1236 ± 20	1231 ± 17	1247 ± 10	1227 ± 16
Porosity/% (*n* = 10–17)	fl/fl	2.20 ± 0.20	2.81 ± 0.50*	2.84 ± 0.48*	2.52 ± 0.35^	3.09 ± 0.52*	2.89 ± 0.52*
	cKO	2.32 ± 0.23	2.55 ± 0.46	2.74 ± 0.43*	2.31 ± 0.22	2.97 ± 0.32^*	2.58 ± 0.25
pMOI/mm^4^ (*n* = 11–17)	fl/fl	0.40 ± 0.04	0.51 ± 0.08*	0.47 ± 0.06*	0.38 ± 0.06	0.48 ± 0.10*	0.45 ± 0.08*
	cKO	0.44 ± 0.07	0.45 ± 0.08	0.49 ± 0.09	0.41 ± 0.07	0.43 ± 0.06	0.40 ± 0.06^
Anterior- Posterior, caliper/mm (*n* = 11–17)	fl/fl	1.25 ± 0.07	1.28 ± 0.09	1.27 ± 0.07	1.23 ± 0.09	1.25 ± 0.11	1.31 ± 0.11
	cKO	1.31 ± 0.12	1.28 ± 0.09	1.29 ± 0.09	1.30 ± 0.09	1.22 ± 0.08	1.30 ± 0.09
C_min_/μm (*n* = 10–17)	fl/fl	634.9 ± 24.2	674.0 ± 52.9	652.1 ± 29.1	626.6 ± 43.6	660.8 ± 56.0	654.7 ± 46.8
	cKO	670.4 ± 50.7	671.9 ± 48.3	685.6 ± 49.1	648.1 ± 31.1	635.1 ± 32.4	640.2 ± 33.9

Data are presented as mean ± SD. *n* = 10–17 mice/group. ^*P* < 0.05 versus control mice, *P < 0.05 versus respective vehicle-treated mice, and #*P* < 0.05 versus respective fl/fl, by three-way ANOVA with factors of DM, genotype, and treatment

**Table 2:** Vertebral (L6) bone structural indices quantified by micro-CT of Control and DM fl/fl and cKO mice treated with PTH or ABL

Vertebral bone structural indices		Control			DM		
		Veh	PTH_100_	ABL_47.5_	Veh	PTH_100_	ABL_47.5_
(BV/TV)/% (*n* = 11–17)	fl/fl	25.64 ± 1.72	26.44 ± 2.10	27.05 ± 3.78	25.68 ± 2.01	31.42 ± 1.65^*	30.69 ± 1.23^*
	cKO	27.05 ± 3.46	25.02 ± 2.65	23.5 ± 2.03*^#^	24.28 ± 1.12^	27.24 ± 2.90*^^#^	26.38 ± 3.06*^^#^
Tb.Th/mm (*n* = 11–16)	fl/fl	0.046 ± 0.001	0.051 ± 0.002*	0.051 ± 0.004*	0.047 ± 0.001	0.054 ± 0.002*^	0.054 ± 0.002*^
	cKO	0.052 ± 0.004^#^	0.05 ± 0.003	0.05 ± 0.005*^#^	0.048 ± 0.002^	0.051 ± 0.003*^#^	0.051 ± 0.004*^#^
Tb.N/mm^-1^ (*n* = 10–17)	fl/fl	6.10 ± 0.36	6.00 ± 0.42	5.92 ± 0.38	5.84 ± 0.34	6.04 ± 0.26	6.04 ± 0.25
	cKO	5.96 ± 0.34	5.65 ± 0.49^#^	5.54 ± 0.32*^#^	5.68 ± 0.13	5.65 ± 0.19^#^	5.59 ± 0.47^#^
Conn.D/mm^-3^ (*n* = 10–16)	fl/fl	273.6 ± 30.9	367.7 ± 35.4*	396.9 ± 61.1*	241.3 ± 21.9	316.9 ± 46.5*^	339.3 ± 65.5*^
	cKO	237.4 ± 26.6	349.8 ± 51.4*	362.8 ± 71.4*	236.3 ± 30.1	304.3 ± 28.3*^	287.4 ± 37*^^#^
SMI (*n* = 11–17)	fl/fl	0.71 ± 0.14	0.88 ± 0.16	0.86 ± 0.26	0.80 ± 0.17	0.32 ± 0.17*^	0.37 ± 0.13*^
	cKO	0.65 ± 0.33	0.89 ± 0.18*	1.08 ± 0.28*	0.88 ± 0.16^	0.62 ± 0.32*^^#^	0.69 ± 0.32*^^#^
Tb.Sp/mm (*n* = 10–17)	fl/fl	0.156 ± 0.01	0.163 ± 0.01	0.168 ± 0.01*	0.164 ± 0.01	0.154 ± 0.01	0.157 ± 0.01^
	cKO	0.160 ± 0.004	0.175 ± 0.02*^#^	0.182 ± 0.01*^#^	0.17 ± 0.004	0.17 ± 0.007^#^	0.175 ± 0.02^#^
Ct.Th/mm (*n* = 10–16)	fl/fl	0.059 ± 0.01	0.059 ± 0.01	0.060 ± 0.01	0.050 ± 0.01	0.058 ± 0.01	0.055 ± 0.01
	cKO	0.063 ± 0.01	0.057 ± 0.01	0.061 ± 0.01	0.053 ± 0.01^	0.057 ± 0.01	0.054 ± 0.01

Data are presented as mean ± SD. *n* = 10–17 mice/group. ^*P* < 0.05 versus C mice, *P < 0.05 versus respective vehicle-treated mice, and #*P* < 0.05 versus respective fl/fl mice, by three-way ANOVA with factors of DM, genotype, and treatment

In cortical bone, DM decreased cortical area (Ct.Ar) and cortical thickness (Ct.Th) and PTH and ABL increased these indices in control fl/fl mice only. Similarly, ABL restored them only in DM fl/fl mice. In contrast, PTH increase Ct.Ar and Ct.Th in both fl/fl and cKO DM mice (Fig. 3d, Fig. S3c, and 3f, Fig. S3b, and Table 1). The effects on cortical architecture resulted in the expected increase in the polar moment of inertia (pMOI) with PTH and ABL, only in fl/fl mice (Fig. 3e and Table 1). All micro-CT indices of femoral and L6 bone are presented in Table 1 and Table 2, respectively. Thus, overall, the bone gain and architectural improvement/restoration induced by PTH or ABL requires the expression of the PTH1R in DMP1-Cre expressing cells.

### Expression of the PTH1R in DMP1-Cre expressing cells is also required for PTH and ABL to improve bone mechanical properties, increase strength and enhance resistance to fracture

**Fig. 4:**
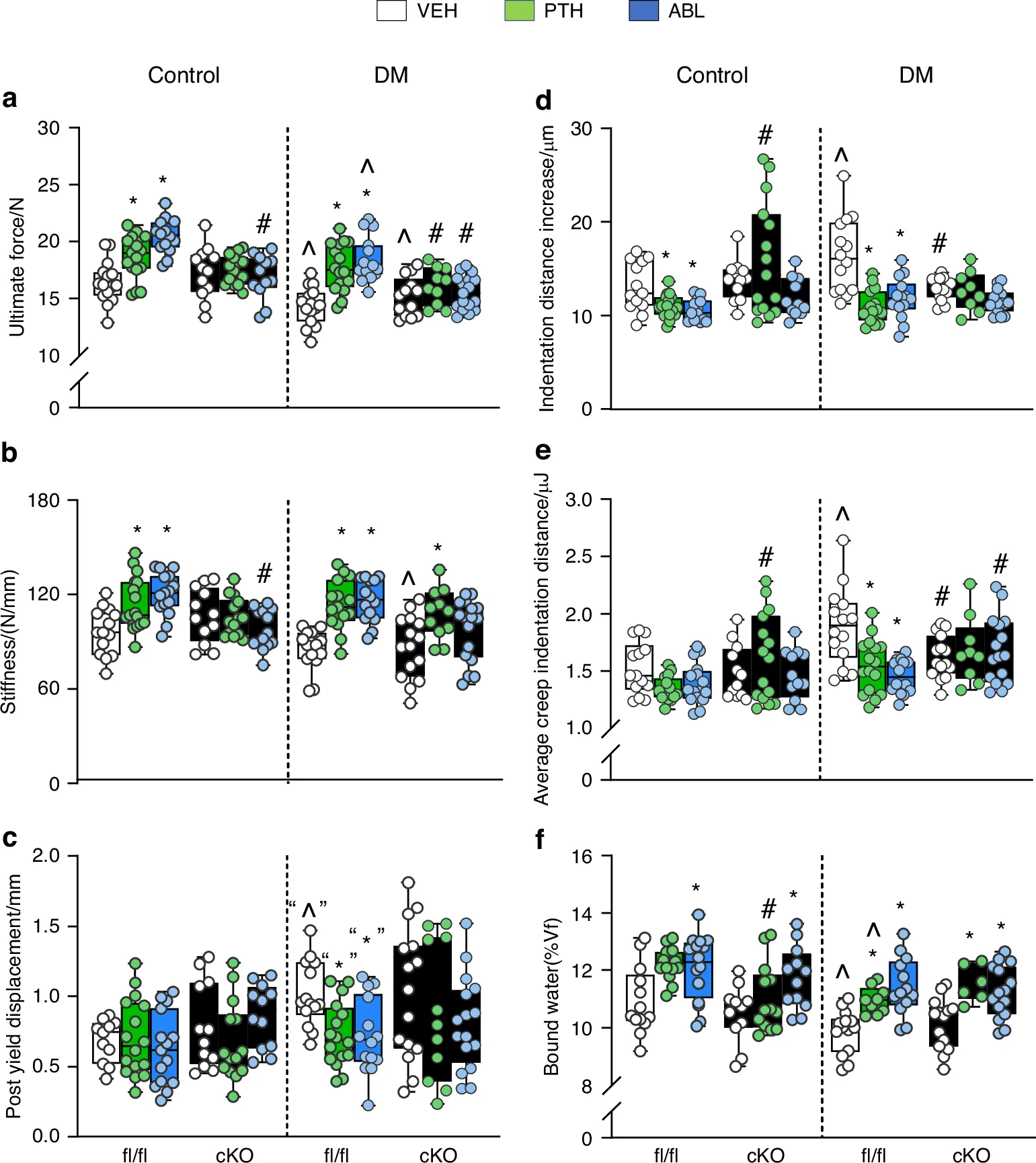
PTH and ABL increased bone strength and improved/corrected the biomechanical properties in fl/fl but not in cKO mice under physiological or diabetic conditions. Bone strength indexes were quantified by 3-point bending test of the femur (**a–c**), cyclic reference point indentation (cRPI) analysis were performed in the tibia (**d, e**), and bound water content was quantified by ^1^H NMR relaxometry analysis of the femur (**f**), of Control and DM mice administered vehicle, PTH or ABL. *n* = 6–17 mice/group. *P < 0.05 versus respective vehicle-treated mice, ^*P* < 0.05 DM versus Control mice, and #*P* < 0.05 versus respective fl/fl mice by three-way ANOVA with factors of DM, genotype, and treatment. ^“^^^”^ unadjusted *P* < 0.05 versus control mice and ^“^*^”^ unadjusted *P* < 0.05 versus DM vehicle-treated fl/fl mice Each dot represents a mouse

We next evaluated the effect of DM and the treatments on bone mechanical and material properties. Femoral 3-point bending testing revealed that DM decreased ultimate force, a measure of strength, in fl/fl and cKO mice; and PTH or ABL increased ultimate force in control mice and restored it in DM mice, only in fl/fl mice but not in cKO mice (Fig. 4a and Table 3). Stiffness, or resistance to deformation, was significantly decreased in DM in cKO mice, PTH/ABL increased it in control and DM fl/fl mice, and PTH also increased it in DM cKO mice (Fig. 4b and Table 3). Further, post-yield displacement (PYD), an index of bone deformation, was trending to higher values in DM fl/fl mice compared to control fl/fl mice; and trending to reversal by PTH or ABL as the adjusted *P* value was < 0.10 and the unadjusted *P* value was < 0.05 (Fig. 4c and Table 3). PYD in cKO mice was not affected by DM or treatments. In contrast to the profound changes in structural properties described above, 3-point-bending analysis did not find major changes in material properties in response to DM or PTH/ABL (Table 3). However, the bone material behavior quantified by cyclic reference point indentation (cRPI) in the tibia showed marked changes induced by DM indicative of bone fragility. Thus, DM elevated Indentation Distance Increase (IDI), Average Creep Indentation Distance (Avg-CID), and Average Energy Dissipated (Avg-ED), in fl/fl mice (Fig. 4d, e and Table 3). These indices were decreased by PTH or ABL. In non-diabetic control fl/fl mice, PTH and ABL decreased IDI (Fig. 4d, e and Table 3). These cRPI indices were not altered by DM in cKO mice. Further, PTH or ABL had no effect in cKO mice either under control or DM conditions.

**Table 3:** Mechanical and material properties of long bones of Control and DM fl/fl and cKO mice treated with PTH or ABL

Mechanical and material properties		Control			DM		
		Veh	PTH_100_	ABL_47.5_	Veh	PTH_100_	ABL_47.5_
3-point bending (femur)							
Structural properties (extrinsic)							
Stiffness/(N/mm) (*n* = 11–17)	fl/fl	96.4 ± 15	114 ± 16.8*	120 ± 13.6*	85.4 ± 12.5	113.7 ± 16*	116.2 ± 13.5*
	cKO	106.8 ± 17	106 ± 12.2	98 ± 12.8^#^	86.6 ± 20^	107.6 ± 16*	98 ± 19.3^#^
Ultimate Force/N (*n* = 10–17)	fl/fl	16.5 ± 1.9	18.7 ± 1.9*	20.5 ± 1.5*	14.4 ± 1.7^	17.8 ± 2.1*	18.4 ± 1.9*^
	cKO	17.3 ± 2.3	17.6 ± 1.3	16.9 ± 2.0^#^	15.2 ± 1.7^	16.1 ± 1.7^#^	15.5 ± 1.5^#^
Post-yield displacement/mm (*n* = 11–16)	fl/fl	0.68 ± 0.15	0.7 ± 0.27	0.62 ± 0.26	1.02 ± 0.23	0.73 ± 0.23	0.72 ± 0.27
	cKO	0.79 ± 0.3	0.68 ± 0.28	0.86 ± 0.23	1.02 ± 0.47	0.89 ± 0.5	0.81 ± 0.33
Work to failure/N·mm (*n* = 11–16)	fl/fl	9.01 ± 3.3	10.9 ± 2.7	11.3 ± 3.8	10.3 ± 1.8	11.0 ± 2.4	11.7 ± 3.6
	cKO	11.4 ± 3.9	10.5 ± 2.5	12.1 ± 2.7	11.0 ± 3.4	10.7 ± 4.1	9.3 ± 2.3
Material properties (intrinsic)							
Ultimate Stress MPa (*n* = 10–17)	fl/fl	153.2 ± 21.5	153.9 ± 17.8	165.9 ± 14.2	149.5 ± 21.5	163.1 ± 19.6	170.3 ± 27.3
	cKO	151.3 ± 14.9	158.8 ± 12.1	145.6 ± 13.3	153.2 ± 15.2	162.3 ± 23	157.8 ± 19.8
Modulus (GPa) (*n* = 11–17)	fl/fl	8.12 ± 1.7	7.61 ± 2.0	8.7 ± 1.4	7.96 ± 1.7	9.01 ± 1.8	8.8 ± 1.9
	cKO	7.98 ± 1.95	7.94 ± 1.98	6.4 ± 1.3#	6.9 ± 1.3	8.77 ± 2.0	8.34 ± 1.7^
Toughness/(N/mm^2^) (*n* = 11–16)	fl/fl	4.4 ± 1.2	4.6 ± 1.1	4.5 ± 1.4	5.2 ± 0.8	4.9 ± 1.0	4.9 ± 1.3
	cKO	4.9 ± 1.1	4.6 ± 1.0	5.2 ± 0.9	5.4 ± 1.6	5.0 ± 1.9	4.6 ± 1.1
Cyclic Reference Point Indentation, cRPI (tibia)							
TID/μm (*n* = 9–17)	fl/fl	78.7 ± 7.2	72.9 ± 4.2	75.8 ± 6.1	79.5 ± 7.2	73.5 ± 3.8	74.4 ± 5.9
	cKO	76.9 ± 7.1	78.1 ± 11.7	72.4 ± 4.2	75.9 ± 4.0	74.8 ± 8.2	79.4 ± 7.9
IDI/μm (*n* = 9–16)	fl/fl	13.1 ± 2.6	11.1 ± 1.3*	10.6 ± 1.2*	16.4 ± 4.0^	11 ± 1.8*	11.8 ± 2.2*
	cKO	13.8 ± 2.1	16.0 ± 6.1^#^	11.9 ± 2.1	12.8 ± 1.2^#^	12.6 ± 2.1^	11.5 ± 1.3
Avg-CID/μm (*n* = 9–16)	fl/fl	1.52 ± 0.2	1.34 ± 0.1	1.39 ± 0.2	1.89 ± 0.3^	1.54 ± 0.2*	1.45 ± 0.2*
	cKO	1.49 ± 0.2	1.61 ± 0.4^#^	1.47 ± 0.2	1.62 ± 0.2^#^	1.68 ± 0.3	1.68 ± 0.3^#^
Avg-ED/μJ (*n* = 10–16)	fl/fl	25.1 ± 4.2	22.24 ± 2.6	22.24 ± 2.6	28.18 ± 4.0^	23.1 ± 2.2*	22.04 ± 2.6*
	cKO	25.16 ± 2.6	24.8 ± 4.7	24.52 ± 3.7	26.27 ± 4.5	24.68 ± 4.3	25.8 ± 3.7^#^
Avg-US/(N/μm) (*n* = 10–16)	fl/fl	0.33 ± 0.03	0.34 ± 0.02	0.34 ± 0.02	0.32 ± 0.02	0.34 ± 0.02	0.33 ± 0.02
	cKO	0.32 ± 0.02	0.34 ± 0.03	0.33 ± 0.03	0.32 ± 0.02	0.32 ± 0.02	0.32 ± 0.01
Avg-LS/(N/μm) (*n* = 10–17)	fl/fl	0.27 ± 0.02	0.28 ± 0.02	0.28 ± 0.01	0.26 ± 0.02	0.28 ± 0.02	0.28 ± 0.01
	cKO	0.26 ± 0.02	0.27 ± 0.02	0.27 ± 0.02	0.26 ± 0.02	0.26 ± 0.03	0.26 ± 0.02
Proton (^1^H) Nuclear Magnetic Resonance, NMR (femur)							
Bound water (%Vf) (*n* = 6–16)	fl/fl	10.93 ± 1.2	12.24 ± 0.57	12.06 ± 1.1*	9.87 ± 0.8^	10.96 ± 0.5*^	11.43 ± 1*
	cKO	10.49 ± 1.0	11.03 ± 1.2^#^	11.73 ± 1.1*	10.14 ± 0.9	11.53 ± 0.6*	11.24 ± 0.9*

Data are presented as mean ± SD. *n* = 6–17 mice/group. ^*P* < 0.05 versus respective Control mice, *P < 0.05 versus respective vehicle-treated mice, and #*P* < 0.05 versus respective fl/fl, by three-way ANOVA with factors of DM, genotype, and treatment

To explore whether DM and/or the treatments cause any matrix-related changes, we next quantified by Proton (^1^H) Nuclear Magnetic Resonance (NMR) relaxometry, the content of loosely bound water in bone (BW), which has been proposed as a critical determinant of bone fragility.^13–15^ BW was lower in DM fl/fl mice but not in cKO mice compared to control mice (Fig. 4f and Table 3). PTH/ABL increased or corrected the BW content in non-diabetic control as well as in DM mice, respectively, of both genotypes, although the PTH effect did not reach statistical significance in control cKO mice.

Overall, our findings are consistent with the notion that strength gains upon PTH or ABL treatment are due to structural changes that require the expression of the PTH1R in DMP1-Cre expressing cells and are unrelated to the increase in BW.

### Actions on DMP1-Cre expressing cells are dispensable for the increase in circulating bone formation and resorption markers, osteoclasts and bone formation on most bone surfaces, but are required for a full response to PTH and ABL on the periosteal and endocortical surfaces

Earlier findings showed that DM impacts bone remodeling, by increasing bone resorption and decreasing bone formation, and that PTH or ABL administration further increase resorption but enhance bone formation leading to net bone gain.^4,9,12^

**Fig. 5:**
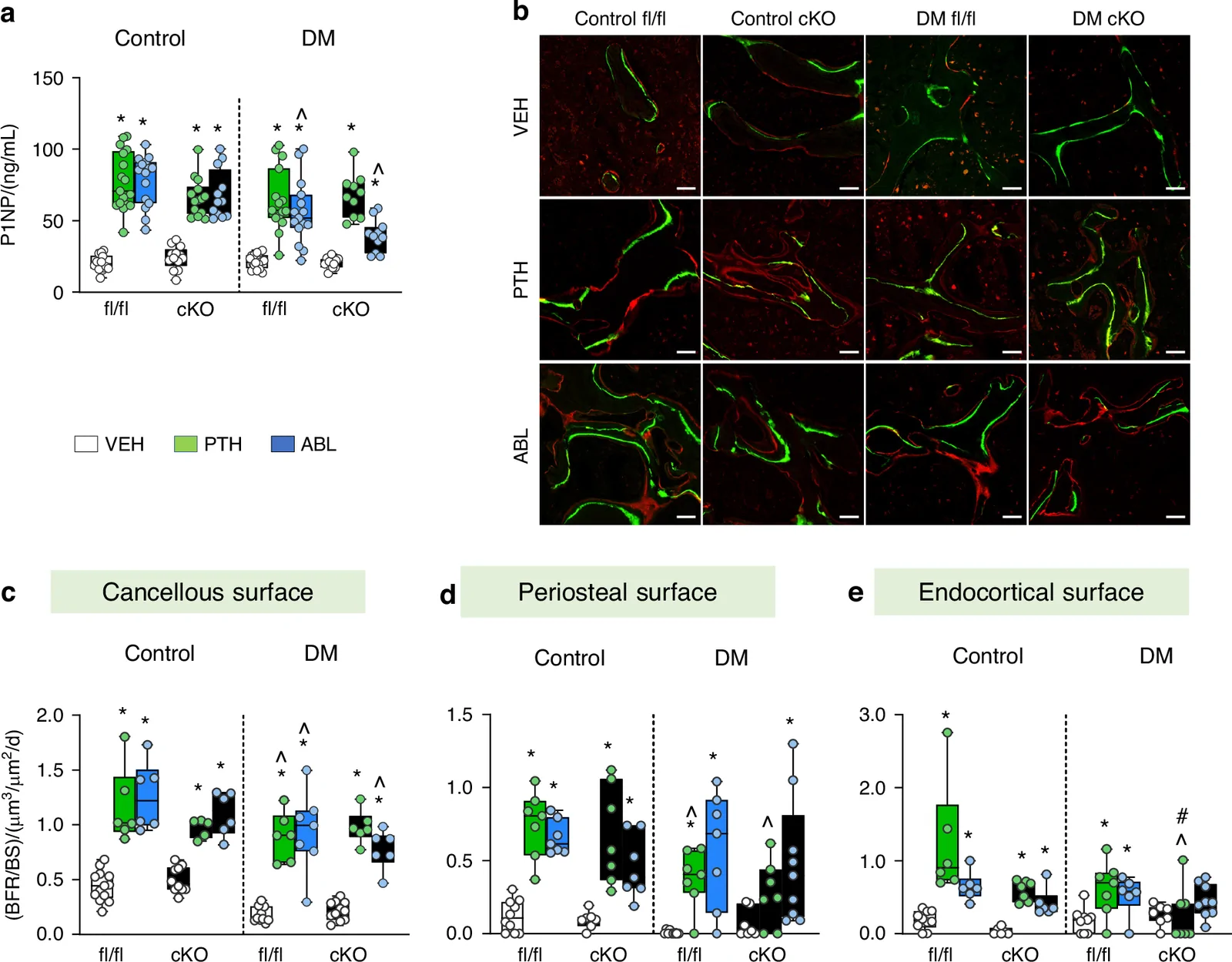
PTH and ABL increased bone formation in control and diabetic fl/fl mice and cKO mice. Serum P1NP (**a**), n10–17. = Dynamic bone histomorphometric analysis of cancellous bone of the distal femur (**b**, **c**), periosteal (**d**), and endocortical (**e**) bone surfaces of the femoral mid-diaphysis. *n* = 5–10mice/group. ^*P* < 0.05 versus control mice, *P < 0.05 versus respective vehicle-treated mice, and #*P* < 0.05 versus respective fl/fl mice, by three-way ANOVA with factors of DM, genotype, and treatment. **b** Scale bars in representative images of dynamic bone formation correspond to 50 micrometers

DM decreased bone formation rate per bone surface (BFR/BS) on the femoral cancellous surface of both fl/fl and cKO mice, as quantified by histomorphometry, due to lower mineral apposition rate (MAR) as well as mineralizing surface per bone surface (MS/BS) (Fig. 5b, c, and Fig. S5a). DM also reduced MS/BS on the periosteal surface in fl/fl mice, although the effect did not reach significancy in cKO mice (Fig. S5b); and DM did not induce detectable reduction in any of the bone formation indices on the endocortical surface (Fig. 5e and Fig. S5c).

Regardless of the genotype, however, PTH or ABL increased serum P1NP in control or DM mice (Fig. 5a). Further, PTH/ABL increased cancellous, periosteal and endocortical BFR/BS in both fl/fl or cKO control mice maintained under physiological conditions (Fig. 5b–e). Under DM conditions, PTH/ABL increased bone formation indices on cancellous surfaces of cKO mice as effectively as in fl/fl mice (Fig. 5b, c, and Fig. S5a). However, on periosteal surfaces, PTH/ABL induced a weaker effect in cKO vs fl/fl DM mice in MS/BS, whereas the ligands exhibited similar effects on MAR and BFR/BS in cKO and fl/fl DM mice (Fig. 5d and Fig. S5b). In addition, whereas PTH and ABL increased bone formation indices on the endocortical surface in fl/fl DM mice, neither ligand was effective in cKO DM mice (Fig. 5e and Fig. S5c).

**Fig. 6:**
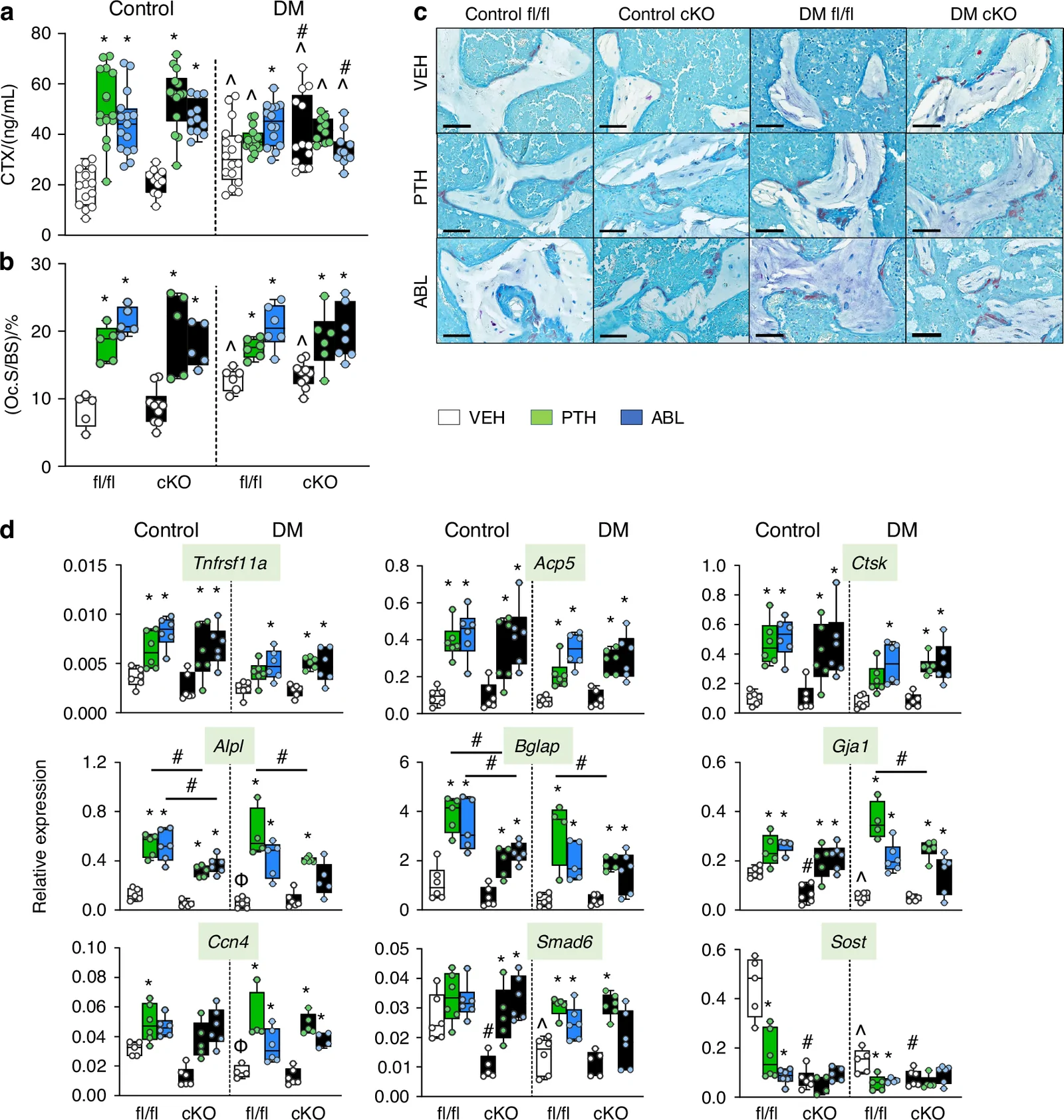
PTH and ABL increased bone resorption in control and diabetic fl/fl and cKO mice. Serum CTX, *n* = 10–16/group (**a**), osteoclast surface/bone surface (Oc.S/BS) in cancellous bone of the distal femur, *n* = 5–10/group (**b**), and representative images (**c**), scale bars correspond to 60 micrometers. Gene expression in tibia (**d**). *n* = 4–6 mice/group. *P < 0.05 versus respective vehicle treated mice, ^*P* < 0.05 versus respective control, #*P* < 0.05 versus respective fl/fl, by three-way ANOVA with factors of DM, genotype, and treatment. Φ*P* < 0.05 versus respective control, by unpaired *t*-test

Regarding resorption, DM mice of either genotype exhibited increased circulating CTX and osteoclast surface on bone (Oc.S/BS). PTH/ABL increased both resorption indices in control mice and further elevated Oc.S/BS in DM mice of both genotypes, whereas ABL also further increased CTX in DM fl/fl mice (Fig. 6a–c).

Overall, PTH and ABL increase bone formation and resorption regardless of whether the PTH1R is expressed in DMP1-Cre expressing cells or not. However, the effect of PTH on bone formation on the endocortical bone surface and of both PTH/ABL on the periosteal surface, were significantly weaker in cKO mice; and neither PTH nor ABL stimulated endocortical bone formation in cKO mice. In contrast, the effects on bone resorption were identical in both genotypes.

### PTH and ABL increased the expression of osteoclast and osteoblast markers and Wnt target genes in both genotypes, but the response on osteoblast markers and Wnt target genes was diminished in cKO mice

Bones from DM fl/fl and cKO mice did not exhibit changes in the expression of osteoclast markers, *Tnfrsf11a* (RANK), *Acp5* (TRAP) and *Ctsk* (CATHEPSIN K), compared to their respective control mice (Fig. 6d). And, PTH/ABL treatment increased osteoclast markers similarly in control and DM mice of both genotypes. Levels of expression of RANKL and OPG were not different among groups (data not shown). In contrast, DM fl/fl bones exhibit lower expression of the osteoblast marker genes *Alpl* (ALKALINE PHOSPHATASE) and *Bglap* (OSTEOCALCIN) and Wnt target genes *Gja1* (CONNEXIN43), *Ccn4* (WISP1) and *Smad6* (SMAD6). Moreover, PTH/ABL treatment increased the expression of the osteoblast markers and the Wnt target gene *Gja1* to a lesser extent in cKO mice compared to fl/fl mice. The expression of the osteocyte gene *Sost* (SCLEROSTIN) was lower in DM fl/fl bones and was decreased by PTH/ABL only in fl/fl mice, confirming the deletion of the PTH1R in osteocytes of cKO mice.

## Discussion

**Fig. 7:**
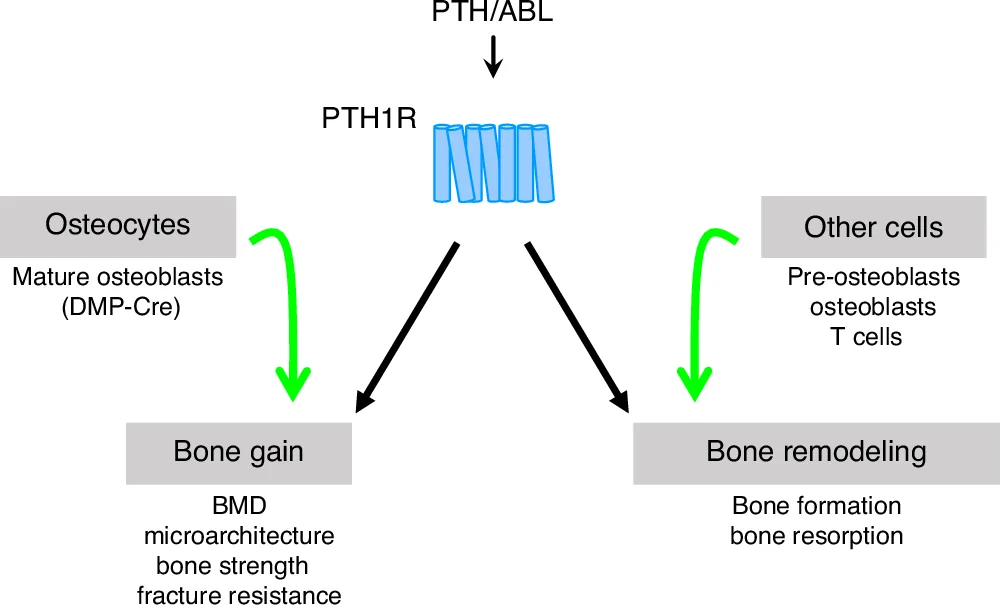
Osteocytes drive bone gain but not bone remodeling induced by PTH and ABL. Signaling downstream of the receptor of parathyroid hormone (PTH1R) exerts two major effects on the skeleton: increases bone remodeling and, when stimulated intermittently, leads to bone gain. Our findings demonstrate dissociation of these two actions by deleting the PTH1R from osteocytes (DMP1-8 kb-Cre expressing cells). We conclude that osteocytes drive bone anabolism induced by PTH/ABL under physiological and diabetic conditions, and that other bone/bone marrow cells are responsible for the increase in bone remodeling

New therapeutic approaches to treat the bone disease due to diabetes are needed, as anti-resorptive treatments do not effectively repair bone microarchitecture or correct bone fragility. We recently showed in pre-clinical studies that bone anabolic agents that activate PTH1R signaling effectively restore the bone lost, increase bone formation, repair microarchitecture, and correct the mechanical and material properties of diabetic bone.^4,9,12^ The PTH1R is expressed in many cell types of the bone microenvironment; however, the identity of the cells mediating the protective effects of PTH and ABL in the frame of diabetes has remained unknown. We report herein that osteocytes, identified as DMP1-Cre expressing cells, are the PTH1R target cells driving the gain in bone mass and strength induced by PTH and ABL; and that, in contrast, the increase in bone remodeling rate (that is, formation that follows resorption) induced by PTH1R activation is mediated by other cells within the bone/bone marrow microenvironment that do not express DMP1-Cre, as PTH/ABL significantly increase remodeling in cKO in the absence of bone gain (Fig. 7).

A limitation of our study is that the DMP1-Cre targets not only all osteocytes, but also mature osteoblasts. However, accumulating evidence demonstrates that deletions of the same genes with DMP1-Cre or the Collagen/Osteocalcin Cre deletors that target preferentially osteoblasts, produce distinct phenotypes demonstrating that DMP1 vs Collagen/Osteocalcin Cres are targeting different cell types.^16^ In addition, the alternative deletor for osteocytes, the Sost-Cre, targets several bone marrow cell populations and only a subpopulation of osteocytes, thus having its own limitations.^17^ Therefore, at present, there is no better Cre deletor to target all osteocytes than the DMP1-Cre mice. Nevertheless, validation of the current findings using a to-be-developed osteocyte-specific Cre deletor is warranted.

Similar to our earlier findings showing that osteocytes are critical for PTH action,^11,18,19^ the current results suggest that DMP1-Cre expressing cells are also the bone target cells mediating the anabolic effects of ABL. This evidence argues against the notion that differences in potency between PTH and ABL result from actions on distinct target cells. Remarkably, the action of both PTH and ABL on DMP1-Cre expressing cells is not essential for their pro-remodeling effects. Therefore, future studies are warranted to reveal the cause underlying the higher potency of ABL compared to PTH, as well as to identify the cells within the bone/bone marrow microenvironment required for the ligands to increase bone remodeling.

Our findings demonstrate dissociation of bone gain from bone remodeling induced by PTH/ABL by deleting the PTH1R from DMP1-Cre expressing cells. It has long been recognized that two major effects of PTH1R activation are to increase the bone remodeling, which “per se” might lead to bone loss,^20,21^ and to induce bone gain when activated intermittently. The results of our study reconcile these two seemingly contradictory facts because they show that bone gain and the increase in bone remodeling are mediated by different cell types, as genetically deleting PTH1R action from DMP1-Cre expressing cells removes the ability of the receptor ligands to induce bone gain, without blocking their ability to increase bone remodeling.

Although bone formation (and resorption) was still enhanced by PTH/ABL in the cKO mice, the degree of the increase in bone formation in some bone surfaces was weaker, which is consistent with our previous findings with PTH.^11^ Specifically, PTH/ABL were less effective in increasing BFR/BS on the endocortical surface of cKO mice and the response on periosteal MAR was absent under DM conditions. Potentially, reduced bone formation indices in cKO mice could have resulted from unwanted deletion of the receptor from osteoblasts, known to be targeted by the DMP1-8kb-Cre-driven deletor.^16^ However, the bone formation responses to PTH or ABL persist in cKO mice, suggesting that the PTH1R is still expressed in osteoblasts and implying that other non-DMP1Cre expressing cells may have contributed to the anabolic actions, presumably through PTH1R.

The findings at the tissue level on bone formation align with the molecular changes on the expression of osteoblast marker genes. Thus, DM reduces *Alpl* and *Bglap* expression whereas PTH and ABL increase it, although at lower magnitude in cKO mice. Further, earlier findings using gene ontology mapping demonstrated that PTH and ABL corrected the transcriptomic changes induced by diabetes of decreased Wnt signaling.^9^ We now show the decreased expression of several Wnt target genes induced by diabetes and the reversal by PTH and ABL. Consistent with the widespread activation of Wnt signaling in several bone cell types, PTH/ABL increased Wnt target gene expression even in cKO mice. However, the effect of PTH/ABL on the Wnt target gene Gja1 (encoding CONNEXIN43), which is expressed at higher levels in osteocytes compared to osteoblasts, was diminished in cKO mice compared to fl/fl mice.

The need of expression of the PTH1R in DMP1-Cre expressing cells for PTH/ABL to induce bone gain demonstrates that these cells drive bone anabolism in response to PTH1R ligands, even in the face of high bone remodeling. Similar response is induced by genetic activation of Wnt signaling in DMP1-Cre expressing cells.^22^ Taken together with the restoration of Wnt signaling by PTH/ABL in the frame of diabetes,^9^ these findings strongly suggest that the underlying mechanism of osteocyte-driven bone gain in response to anabolic treatments depends on Wnt activation. Future studies are warranted to validate this premise.

The increase in fracture risk in diabetes is not fully captured by changes in BMD. Indeed, some patients exhibit bone fragility with normal or even increased BMD suggesting that diabetes alters bone tissue material properties.^23,24^ Our study together with previously reported evidence from us and others,^9,25^ demonstrates that, despite the limitations of every model, the high fat diet/STZ combination in mice is a reliable model of T2-DM because it uses skeletally mature mice, mimics the adult onset of diabetes, and closely mirrors the course of the bone disease induced by diabetes in humans. Our results also provide information about the role of DMP1-Cre expressing cells in determining bone mechanical properties. The bone structural (extrinsic) properties quantified by 3-point bending were altered by diabetes and these changes were associated with marked effects on the bone material behavior evaluated by bone indentation (cRPI), indicative of bone fragility. PTH/ABL corrected both structural and behavioral properties in a manner that depends on the presence of the PTH1R in DMP1-Cre expressing cells, demonstrating the crucial role of these cells in bone strength and resistance to fracture.

The content of loosely bound bone water (BW) plays a pivotal role in maintaining bone toughness^26,27^ and it has been shown to be a determinant of bone fragility in preclinical murine models as well as in clinical settings.^13–15^ Consistently, we found that BW content was lower in diabetic bones of both genotypes. However, in contrast to the bone fragility indices mentioned above that are reversed by PTH/ABL only in fl/fl mice but not in cKO mice, the decreased BW content in diabetic bone was reversed by anabolic treatments regardless of the presence or not of the PTH1R expression in DMP1-Cre expressing cells. These findings are consistent with the notion that strength gains upon PTH or ABL treatments are due to structural changes that require the expression of the PTH1R in DMP1-Cre expressing cells and are unrelated to the increase in BW. Water retention in bone is controlled by the proteoglycan content of glycosaminoglycans (GAGs).^28^ Therefore, future studies are needed to ascertain whether GAGs levels are altered by PTH/ABL in a manner that is also independent of the receptor expression in osteocytes.

In closing, our findings demonstrate that DMP1-Cre expressing cells are the PTH1R target cells driving the gain in bone mass and strength induced by PTH and ABL and reveal the dissociation of bone gain from bone remodeling induced by this pathway by deleting the PTH1R from DMP1-Cre expressing cells.

## Materials and methods

### Animals and experimental design

Mice with conditional deletion of parathyroid hormone receptor 1 (PTH1R) in osteocytes and some mature osteoblasts (cKO) were generated by crossing PTH1R flox/flox mice (fl/fl)^29^ with DMP1-8kb-Cre mice,^30^ as published earlier.^11^ cKO mice were crossed with DMP1-GFP mice in which osteocytes are labeled with green fluorescent protein (GFP), GFP^+^ cells (osteocytes) were separated from GFP- cells (osteoblasts and other cells) by sorting bone cell preparations, and the expression of PTH1R was quantified. GFP^+^ cells, but not GFP^-^ cells, exhibited lower expression of PTH1R in cKO mice,^11^ demonstrating selective deletion of the PTH1R in osteocytes. It is well documented for rodents that males are more susceptible to develop DM than females, although the causes remain uncertain. We validated this fact in previous studies showing that male mice exhibited high blood glucose, impaired glucose tolerance, and bone loss readily after STZ injections, but female mice did not.^9,12^ Thus, we elected to study male mice, following the guidance of the diabetes field. Twelve-week-old male mice were housed ≤ 5 mice/cage, received water *ad-libitum*, and were exposed to 12 h light/dark cycles. Mice were assigned to treatment groups according to standard stratified randomization methods, as follows. Mice within each genotype were assigned into diet groups at t0 by sorting on total body BMD, followed by randomizing each pair of mice from these sorted lists between the two diet groups to achieve random groups balanced on total BMD. (Fig. 1a) and fed throughout the experiment a low-fat diet (LFD) or high-fat diet (HFD) with 10 or 60 Kcal% fat, respectively (Research Diets Inc. D12450J and D12492). Four weeks after initiating the diets (t1), DM was induced in HFD-fed mice by 5 daily injections of STZ (45 mg/kg i.p. in 100 mmol/L citrate buffer, pH 4.6) while control LFD-fed mice received citrate buffer (Control), as published.^9,25^ Four weeks after STZ injections (t2), DM was confirmed by blood glucose values > 250 mg/dL. Four weeks later at t3, mice within each group (control or DM of each genotype) were again sorted, now each triplet of mice from these sorted lists was randomized by total body BMD between the three treatment groups, followed by confirmation by a *t*-test that the means corresponding to blood glucose level and body weight are not different between the groups. Mice received daily s.c. injections of vehicle (0.9% saline, 0.01 mmol/L β-Mercaptoethanol, 0.01% acetic acid), PTH (100 µg/kg/d, Bachem, Torrance, CA), or ABL (47.5 µg/kg/d, Radius Pharmaceutical, Boston, MA), for 28 d (t4), followed by euthanasia and tissue procurement (Fig. 1a). All analyses corresponding to the skeletal phenotype were performed in a blinded fashion.

### Blood glucose measurements

Blood for the glucose measurements was collected from the facial vein of 5-h fasted mice using AlphaTrak 2 Blood Glucose Monitoring System (Zoetis Florham Park, NJ, USA). Average of 2 measurements recorded using 2 different monitors are reported.

### Bone turnover markers

Blood was collected from the facial vein of 5-h fasted mice. Procollagen type 1 N-terminal propeptide (P1NP) and C-telopeptide fragments of type I collagen (CTX) (RatLaps, Immunodiagnostic Systems Inc., Fountain Hills, AZ, USA) were quantified following manufacturer’s instructions.

### Bone mineral density measurements

Longitudinal BMD measurements were performed at t0, t1, t2, t3 and t4 by dual-energy x-ray absorptiometry (DEXA) using a PIXImus densitometer (G.E. Medical Systems, Lunar Division, Madison, WI), as published.^9^ Percent changes were calculated with the formula: [(t4-t3)/t3] x 100.

### Bone microarchitecture analysis

Right femurs and L6 vertebrae were dissected, cleaned off soft tissue, and stored in phosphate buffered saline (PBS) at pH 7.4 at −20 °C. Bones were scanned while immersed in PBS by micro-computed tomography (micro-CT).

Femoral analysis was performed using a μCT50 scanner (Scanco Medical AG, Brüttisellen, Switzerland), as published,^31^ under the following parameters: E = 70 kVp, I = 114 uA, 0.5 mm Al filter, integration time = 300 ms, 1 162 samples per 1 000 projections per rotation, at an isotropic voxel size of 6 µm. A beam hardening calibration factor for 1 200 mg hydroxyapatite (HA) was applied to each scan acquisition. The ROI for the femoral distal cancellous bone analysis included the proximal 501 slices (axial length = 3.0 mm) beginning 35 slices (0.21 mm) away from the growth plate to avoid the primary and secondary spongiosa. Cortical porosity (Ct.Po) was quantified by fitting the inner and outer contours to the endosteal and periosteal surfaces, such that Ct.Po was equal to (1–BV/TV), where BV is segmented volume of bone tissue and TV is the total volume defined by the two contours. Femoral mid-diaphysis cortical analysis was performed for all 310 acquired slices at the calculated femoral midpoint (ROI length = 1.86 mm).

L6 analysis was performed using a viva CT80 (Scanco Medical, Switzerland), with E = 70 kVp, I = 114 uA, integration time = 200 ms, at an isotropic voxel size of 10.4 µm. For the trabecular analysis a Gaussian filter (sigma = 0.8, support = 1) was applied and a threshold range of 417.3–2 229.3 mg HA/cm^3^ was used. L6 cancellous bone measurements were performed using ROI spanning from the upper to the lower cartilaginous end plates excluding primary spongiosa. L6 cortical bone analysis was performed in a ROI of 10 slices starting 10 slices away from the point of attachment of the first spinous process to the vertebral body towards the caudal growth plate.^32^ All nomenclature, symbols, and units adhered to guidelines in the literature.^33^

### Bone mechanical and material properties

#### Three-point bending

Following micro-CT scanning, femurs were loaded to failure at 3 mm/min in 3-point bending (Dynamight 8841, Instron, Norwood, MA, USA). The span was fixed at 8 mm. With the medial side facing forward, the mid-point was centered below the loading actuator such that the anterior side was in tension during bending. The force from a 100 N load cell (Honeywell, Morristown, NJ, USA) and displacement from the Instron actuator via the linear variable differential transformer (LVDT), were recorded at 50 Hz for each bending test. The resulting force-displacement data was processed using custom script in Matlab r2024 (Mathworks, Natick, MA) to calculate the strength measurements such as yield force (identified by the 0.2% offset method) and ultimate force. To estimate material strength, yield force and ultimate force (N) were converted yield stress and ultimate stress (MPa) using the flexural formula from Euler-Bernoulli beam theory (force x span/4 x c_min_/I_min_). Section modulus (I_min_/c_min_) was derived from the micro-CT evaluations of the mid-diaphysis.^34^ Other mechanical properties included stiffness (slope of initial linear force vs. displacement curve), post-yield displacement (displacement at fracture minus yield displacement, work-to-fracture (area under the force vs. displacement curve, Wf), modulus (stiffness x span^3^/I_min_/48), and toughness (3 x Wf/cortical bone area/span).

#### Cyclic reference point indentation (cRPI)

The microindentation was performed on the anterio-lateral surface of the intact left tibia using the BioDent instrument (Active Life Scientific, Santa Barbara, CA, USA) with a BP2 test probe (90-degree cono-spherical tip geometry with ≤ 5 μm radius), as described in.^35^ While the bone irrigated with water, indents were perfomred at 8 sites spanning the tibia-fibular junction, each spaced 0.75–0.80 mm apart. During each cycle of loading (10 cycles at 2 Hz), the maximum force was 6 N with a dwell time of 167 ms between loading and unloading. The resulting force vs. data was process as previously described by us^35^ to determine the total indentation distance (TID), indentation distance increase (IDI), average values of energy dissipated (Avg-ED), creep indentation distance (Avg-CID), loading slope (Avg-LS), and unloading slope (Avg-US).

#### ^1^H Nuclear Magnetic Resonance (NMR) relaxometry

After thawing to room temperature, each femur was removed from PBS, dabbed dry and weighed in air (wet mass) and while submerged in DI water to determine the volume of the bone [(wet mass – submerged mass)/density of water]. The femur was then sealed in a 10 mm NMR glass tube alongside a known volume of water (21.2 µL) in a reference microsphere. This tube was next inserted into a custom, low-proton, loop-gap style radiofrequency (RF) coil within a 4.7-T horizontal magnet resonance scanner (Varian Medical Systems, Santa Clara, CA). Following the acquisition of Carr-Purcell-Meiboom-Gill (CPMG) measurements,^36^ a T2 spectrum was generated as detailed earlier.^37^ By including the reference microsphere, the signal intensity under the bound water peak (T2 = 158 to 0.68 µs) was converted to volume based on the mean signal intensity of the reference peak, which had the longest T2 (~2–3 s). Bound water was expressed as a volume fraction based on the femur volume.

### Bone histomorphometric analysis

For dynamic histomorphometric analysis, calcein green (30 mg/kg bw) and alizarin complexone (50 mg/kg bw) were i.p. injected 10 and 3 d prior to euthanasia, respectively. Periosteal and endosteal bone formation measurements were done on the entire surfaces of thick cross-sections at the femoral mid-diaphysis, prepared using a diamond-embedded wire saw (Histosaw, Delaware Diamond Knives, Wilmington, DE, USA) and grounded to a final thickness of 80 µm.^9^ For cancellous bone measurements, thin (5 µm) longitudinal sections of the distal half of the femur were prepared; and bone formation and resorption indices were measured starting 400 μm from the distal growth plate and ending 1 200–1 600 μm proximal to the distal growth plate, respectively. For osteoclast measurements, sections were stained for TRAPase and counterstained with Toluidine blue,^9^ and surfaces covered by TRAPase^+^ cells with three or more nuclei were quantified. Histomorphometric analyses were quantified using OsteoMeasure High Resolution Digital Video System (OsteoMetrics, Decatur, GA) interfaced to an Olympus BX53 fluorescence microscope (Olympus America Inc., Center Valley, PA).^9^ Terminology follows the recommendations of the American Society for Bone and Mineral Research.^38^

### RNA isolation and gene expression analysis

Tibias were dissected and marrow content separated by centrifugation. Bones were snap-frozen in liquid nitrogen and stored at –80 °C. RNA was extracted with TRIzol (Invitrogen) and cDNA was synthesized using the high-capacity cDNA reverse transcription kit (Applied Biosystems). For qPCR, primers and probes were designed using the Assay Design Center (Roche Applied Science) or were commercially available (Applied Biosystems). Analysis was carried out using the QuantStudio 6 pro system (Applied Biosystems, Foster City, CA, USA). Relative mRNA expression levels were normalized to the housekeeping β-actin by using the 2 to the power of negative ∆Ct method.^9^

### Statistical analysis

Statistical analysis was performed using SAS 9.4 (SAS Cary, NC, US). Outcomes at t4 were analyzed by three-way ANOVA models with factors of DM, genotype, and treatment. Outcomes over multiple timepoints were analyzed by Repeated Measures models with effects of DM, genotype, and timepoint. Treatment was not a factor in the Repeated Measure models because at t4 in the longitudinal analysis only data from vehicle-administered mice are included. Comparisons between individual timepoints were not of interest and were not made. For all models, residuals were inspected for normality and homoscedasticity. Data transformations were applied to meet these assumptions when needed. The False Discovery Rate (FDR) adjustment method was used to adjust the *P*-values of planned comparisons for multiplicity. Values outside of mean ± 2 SD were considered outliers and excluded from the analysis.

### Study approval

All animal procedures were approved by the Institutional Animal Care and Use Committee at University of Arkansas for Medical Sciences and the Central Arkansas Veterans Healthcare System, and animal care was carried out in accordance with institutional guidelines.

## Supplementary information

Supplementary Figures

Supplementary Figure Legends

Supplementary Material List

Raw data for Figure 1b and supplementary figure 1d Glucose concentration timepoints

Raw data for Figure 1c and supplementary figure 1e body change timepoints

Raw data for Figure 2a and supplementary figure 1a Total BMD timepoints

Raw data for Figure 2b and supplementary figure 1b Femoral BMD timepoints

Raw data for Figure 2c and supplementary figure 1c Spinal BMD timepoints

Raw data for Figure 2d Percent Change Total BMD

Raw data for Figure 2 Percent Change femoral BMD

Raw data for Figure 2f Percent change spinal BMD

## Data Availability

All data associated with this study are available upon request.

## References

[1] Sun, H. et al. IDF Diabetes Atlas: Global, regional and country-level diabetes prevalence estimates for 2021 and projections for 2045. *Diab. Res. Clin. Pract.* **183**, 109119 (2022). 10.1016/j.diabres.2021.109119 PMC11057359 34879977

[2] Hofbauer, L. C. et al. Bone fragility in diabetes: novel concepts and clinical implications. *Lancet Diab. Endocrinol.* **10**, 207–220 (2022). 10.1016/S2213-8587(21)00347-8 35101185

[3] Shen, Q. & Ma, Y. Impact of diabetes mellitus on risk of major complications after hip fracture: a systematic review and meta-analysis. *Diabetol. Metab. Syndr.* **14**, 51 (2022). 10.1186/s13098-022-00821-0 PMC9003957 35414035

[4] Marino, S. & Bellido, T. PTH receptor signalling, osteocytes and bone disease induced by diabetes mellitus. *Nat. Rev. Endocrinol.* **20**, 661–672 (2024). 10.1038/s41574-024-01014-7 PMC13001153 39020007

[5] Dhaliwal, R. et al. Abaloparatide in postmenopausal women with osteoporosis and type 2 diabetes: a post hoc analysis of the ACTIVE study. *JBMR* **4**, e10346 (2020). 10.1002/jbm4.10346 PMC7117849 32258965

[6] Schwartz, A. V. et al. Teriparatide in patients with osteoporosis and type 2 diabetes. *Bone* **91**, 152–158 (2016). 10.1016/j.bone.2016.06.017 27374026

[7] Munekawa, C. et al. Effect of teriparatide on bone mineral density and trabecular bone score in type 2 diabetic patients with osteoporosis: a retrospective cohort study. *Medicina (Kaunas)* **58**, 481 (2022). 10.3390/medicina58040481 PMC9030978 35454320

[8] Sølling, A. S. K., Harsløf, T. & Langdahl, B. The clinical potential of romosozumab for the prevention of fractures in postmenopausal women with osteoporosis. *Therapeutic Adv. Musculoskelet. Dis.* **10**, 105–115 (2018). 10.1177/1759720X18775936 PMC6009094 29942362

[9] Marino, S. et al. Reversal of the diabetic bone signature with anabolic therapies in mice. *Bone Res* **11**, 19 (2023). 10.1038/s41413-023-00261-0 PMC10115794 37076478

[10] O’Brien, C. A. et al. Control of bone mass and remodeling by PTH receptor signaling in osteocytes. *PLoS One* **3**, e2942 (2008). 10.1371/journal.pone.0002942 PMC2491588 18698360

[11] Delgado-Calle, J. et al. Control of bone anabolism in response to mechanical loading and PTH by distinct mechanisms downstream of the PTH receptor. *J. Bone Miner. Res.* **32**, 522–535 (2017). 10.1002/jbmr.3011 PMC8502039 27704638

[12] Marino, S. et al. Abaloparatide is more potent than teriparatide in restoring bone mass and strength in type 1 diabetic male mice. *Bone*, **181**, 117042 (2024). 10.1016/j.bone.2024.117042 PMC13147481 38360197

[13] Samuel, J., Sinha, D., Zhao, J. C. & Wang, X. Water residing in small ultrastructural spaces plays a critical role in the mechanical behavior of bone. *Bone* **59**, 199–206 (2014). 10.1016/j.bone.2013.11.018 PMC3877214 24291421

[14] Nyman, J. S. et al. Partial removal of pore and loosely bound water by low-energy drying decreases cortical bone toughness in young and old donors. *J. Mech. Behav. Biomed. Mater.* **22**, 136–145 (2013). 10.1016/j.jmbbm.2012.08.013 PMC3655090 23631897

[15] Nyman, J. S. et al. Toward the use of MRI measurements of bound and pore water in fracture risk assessment. *Bone* **176**, 116863 (2023). 10.1016/j.bone.2023.116863 PMC10528882 37527697

[16] Kalajzic, I. et al. In vitro and in vivo approaches to study osteocyte biology. *Bone* **54**, 296–306 (2013). 10.1016/j.bone.2012.09.040 PMC3566324 23072918

[17] Fu, Q. et al. Reduced osteoprotegerin expression by osteocytes may contribute to rebound resorption after denosumab discontinuation. *JCI Insight* **8**, e167790 (2023). 10.1172/jci.insight.167790 PMC10561722 37581932

[18] Bellido, T., Saini, V. & Pajevic, P. D. Effects of PTH on osteocyte function. *Bone* **54**, 250–257 (2013). 10.1016/j.bone.2012.09.016 PMC3552098 23017659

[19] Powell, W. F. et al. Targeted ablation of the PTH/PTHrP receptor in osteocytes impairs bone structure and homeostatic calcemic responses. *J. Endocrinol.* **209**, 21–32 (2011). 10.1530/JOE-10-0308 PMC3783949 21220409

[20] Parfitt, A. M. in *Osteoporosis* 315–329 (Academic Press, 1996).

[21] Parfitt, A. M. High bone turnover is intrinsically harmful: two paths to a similar conclusion. The Parfitt view. *J. Bone Miner. Res*. **17**, 1558–1559 (2002). 10.1359/jbmr.2002.17.8.1558 12162510

[22] Tu, X. et al. Osteocytes mediate the anabolic actions of canonical Wnt/b-catenin signaling in bone. *Proc. Natl. Acad. Sci. USA* **112**, E478–E486 (2015). 10.1073/pnas.1409857112 PMC4321271 25605937

[23] Lekkala, S., Taylor, E. A., Hunt, H. B. & Donnelly, E. Effects of diabetes on bone material properties. *Curr. Osteoporos. Rep.* **17**, 455–464 (2019). 10.1007/s11914-019-00538-6 PMC6986388 31713179

[24] Qian, W. et al. Bone intrinsic material and compositional properties in postmenopausal women diagnosed with long-term Type-1 diabetes. *Bone* **174**, 116832 (2023). 10.1016/j.bone.2023.116832 PMC11302406 37385427

[25] Eckhardt, B. A. et al. Accelerated osteocyte senescence and skeletal fragility in mice with type 2 diabetes. *JCI Insight* **5**, e135236 (2020). 10.1172/jci.insight.135236 PMC7253018 32267250

[26] Wang, X., Xu, H., Huang, Y., Gu, S. & Jiang, J. X. Coupling effect of water and proteoglycans on the in situ toughness of bone. *J. Bone Miner. Res.* **31**, 1026–1029 (2016). 10.1002/jbmr.2774 PMC4862903 26709950

[27] Rao, G. et al. Reactive oxygen species mediate high glucose-induced heparanase-1 production and heparan sulphate proteoglycan degradation in human and rat endothelial cells: a potential role in the pathogenesis of atherosclerosis. *Diabetologia* **54**, 1527–1538 (2011). 10.1007/s00125-011-2110-z 21424539

[28] Piccoli, A. et al. Sclerostin regulation, microarchitecture, and advanced glycation end-products in the bone of elderly women with type 2 diabetes. *J. Bone Miner. Res.* **35**, 2415–2422 (2020). 10.1002/jbmr.4153 PMC8143610 32777114

[29] Kobayashi, T. et al. PTHrP and Indian hedgehog control differentiation of growth plate chondrocytes at multiple steps. *Development* **129**, 2977–2986 (2002). 10.1242/dev.129.12.2977 12050144

[30] Bivi, N. et al. Cell autonomous requirement of connexin 43 for osteocyte survival: consequences for endocortical resorption and periosteal bone formation. *J. Bone Miner. Res.* **27**, 374–389 (2012). 10.1002/jbmr.548 PMC3271138 22028311

[31] Creecy, A. et al. Low bone toughness in the TallyHO model of juvenile type 2 diabetes does not worsen with age. *Bone* **110**, 204–214 (2018). 10.1016/j.bone.2018.02.005 PMC5878744 29438824

[32] Sato, A. Y. et al. Protection from glucocorticoid-induced osteoporosis by anti-catabolic signaling in the absence of Sost/sclerostin. *J. Bone Miner. Res.* **31**, 1791–1802 (2016). 10.1002/jbmr.2869 PMC8499032 27163932

[33] Bouxsein, M. L. et al. Guidelines for assessment of bone microstructure in rodents using micro-computed tomography. *J. Bone Miner. Res.* **25**, 1468–1486 (2010). 10.1002/jbmr.141 20533309

[34] Creecy, A. et al. The age-related decrease in material properties of BALB/c mouse long bones involves alterations to the extracellular matrix. *Bone* **130**, 115126 (2020). 10.1016/j.bone.2019.115126 PMC6885131 31678497

[35] Ahmed, R. et al. Identifying bone matrix impairments in a mouse model of neurofibromatosis type 1 (NF1) by clinically translatable techniques. *J. Bone Miner. Res.* **37**, 1603–1621 (2022). 10.1002/jbmr.4633 PMC9378557 35690920

[36] Horch, R. A., Nyman, J. S., Gochberg, D. F., Dortch, R. D. & Does, M. D. Characterization of 1H NMR signal in human cortical bone for magnetic resonance imaging. *Magn. Reson Med*. **64**, 680–687 (2010). 10.1002/mrm.22459 PMC2933073 20806375

[37] Lane, N. E. et al. Inhibition of vascular endothelial growth factor in young adult mice causes low bone blood flow and bone strength with no effect on bone mass in trabecular regions. *Bone Rep.* **10**, 100210 (2019). 10.1016/j.bonr.2019.100210 PMC6535464 31193542

[38] Dempster, D. W. et al. Standardized nomenclature, symbols, and units for bone histomorphometry: A 2012 update of the report of the ASBMR Histomorphometry Nomenclature Committee. *J. Bone Miner. Res*. **28**, 2–17 (2013). 10.1002/jbmr.1805 PMC3672237 23197339

