# Supplementary material for: DMP1-Cre expressing cells mediate the gain in bone mass and strength, but not the increase in bone remodeling, induced by ligands of the parathyroid hormone receptor
Source: Bone Res. 2026 Jul 30;14:77. doi: 10.1038/s41413-026-00555-z (PMC13424361; doi:10.1038/s41413-026-00555-z)

Supplementary Figure 1

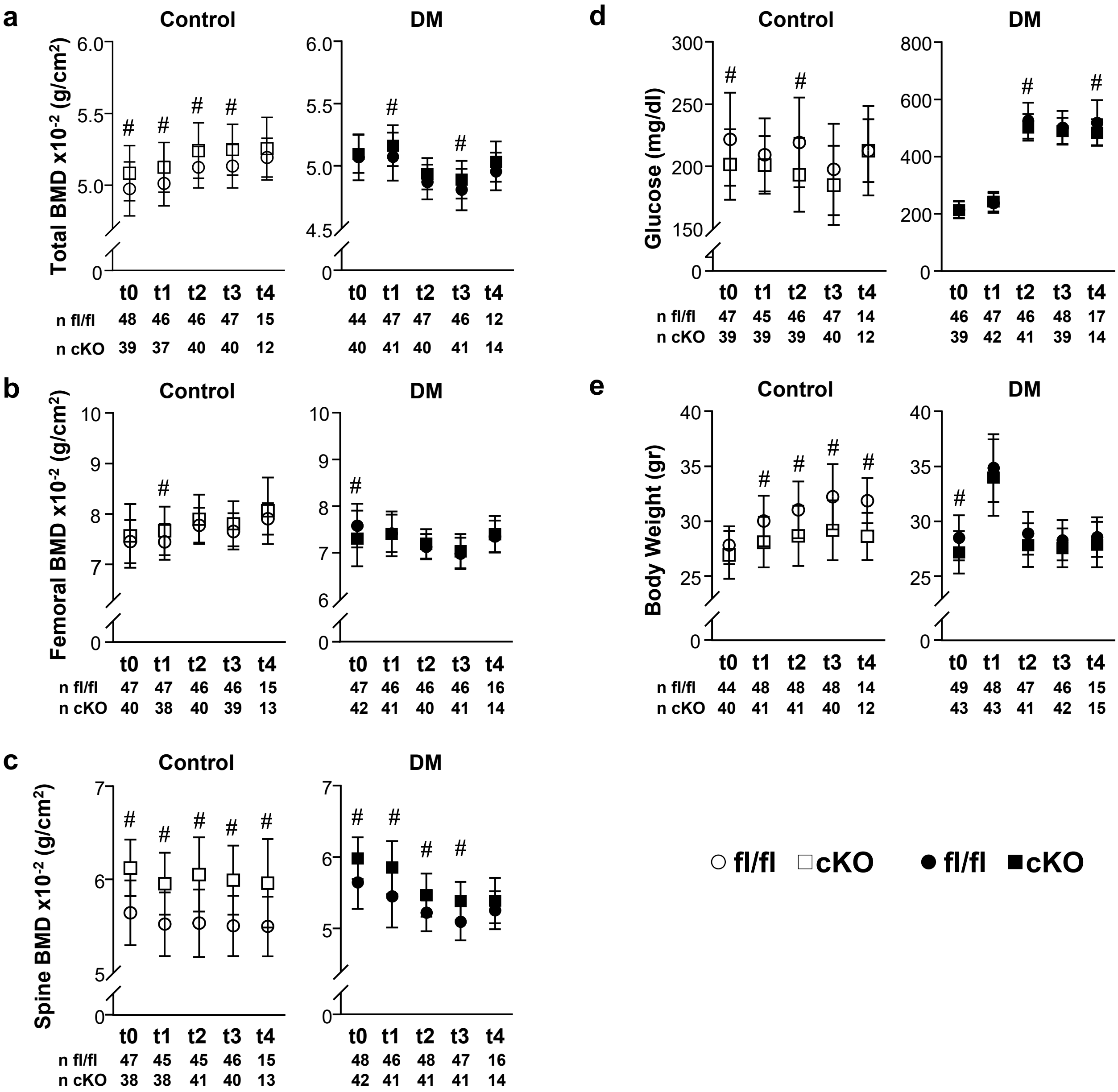

Supplementary Figure 2

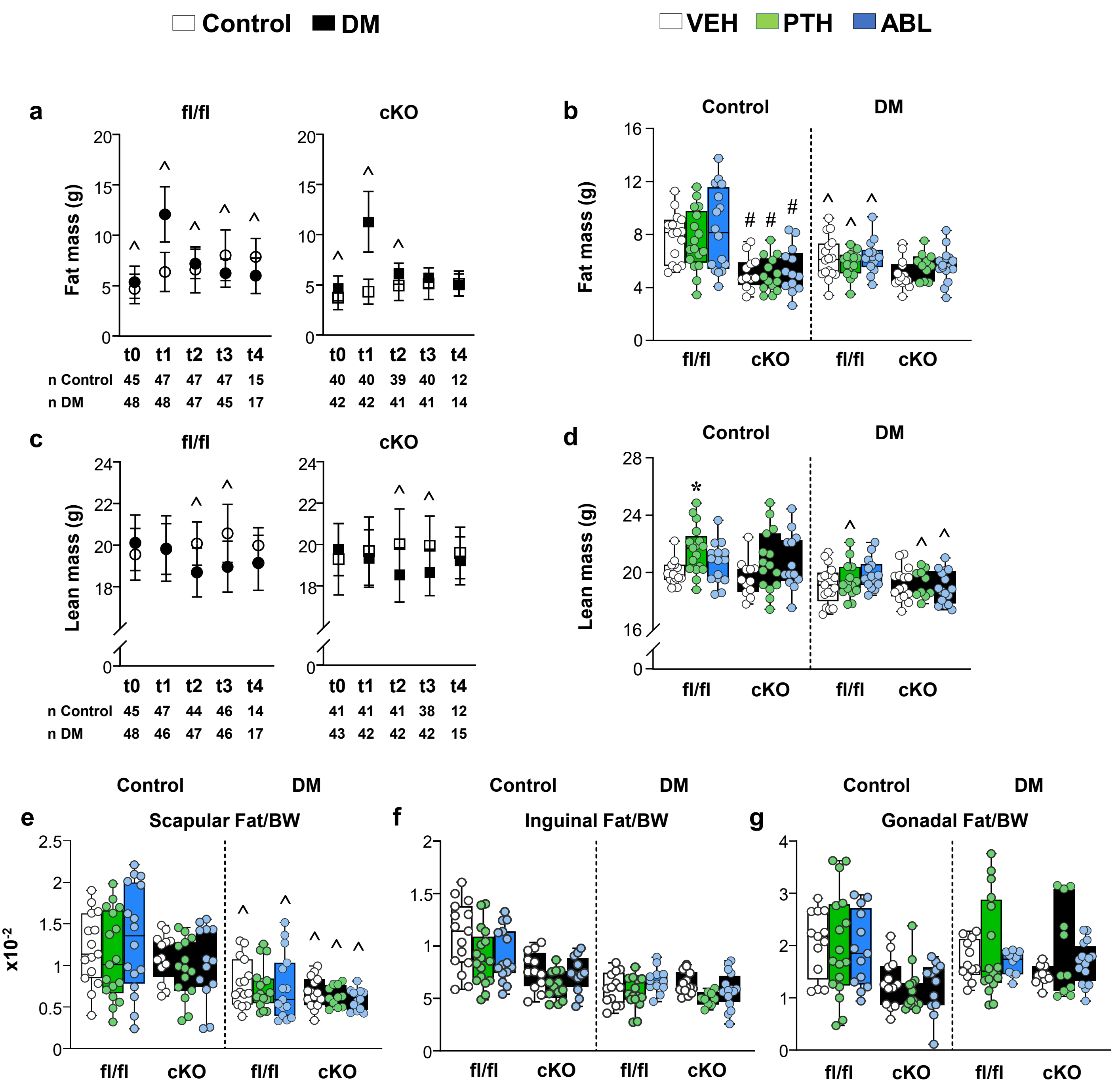

Supplementary Figure 3

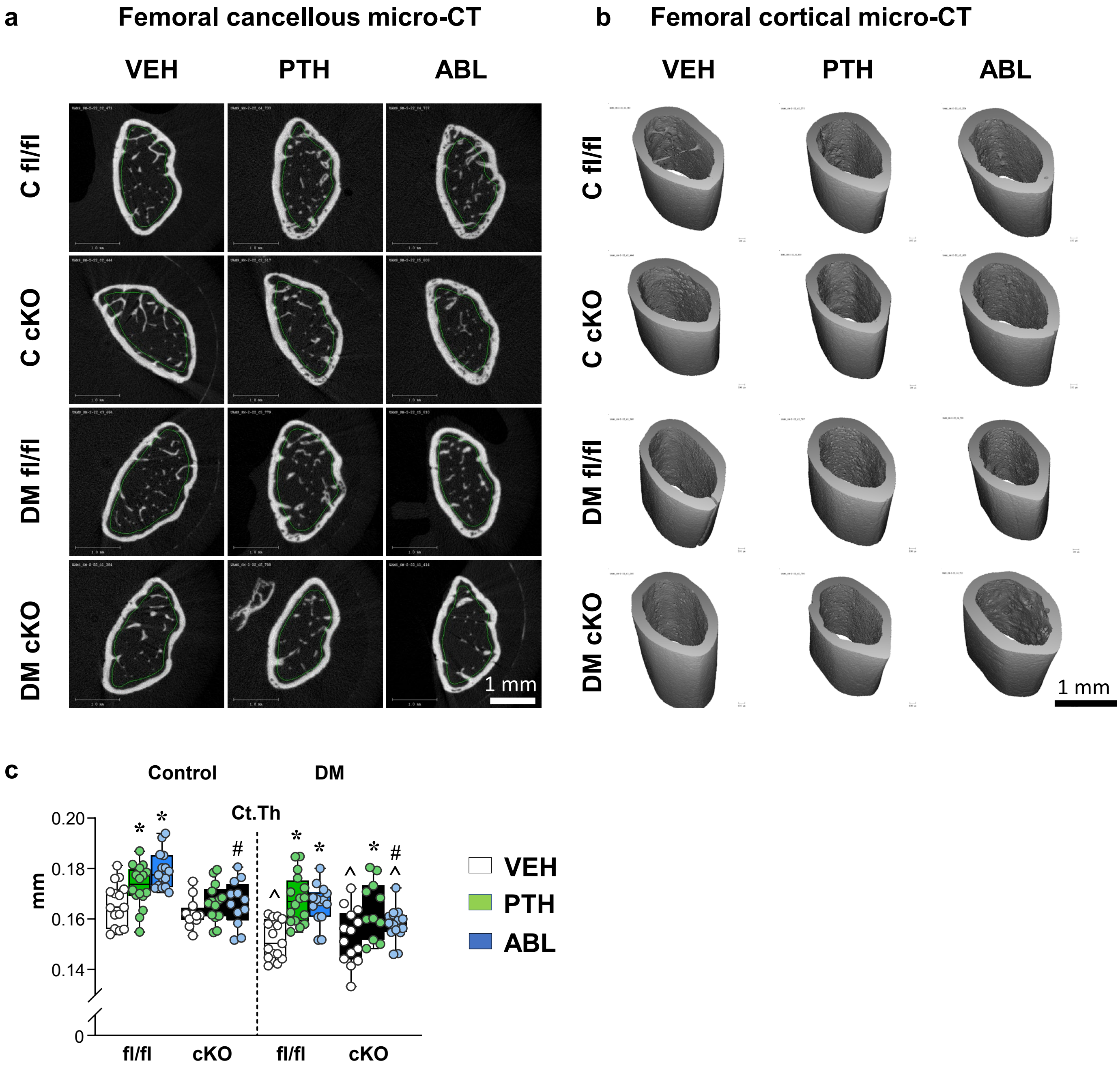

Supplementary Figure 4

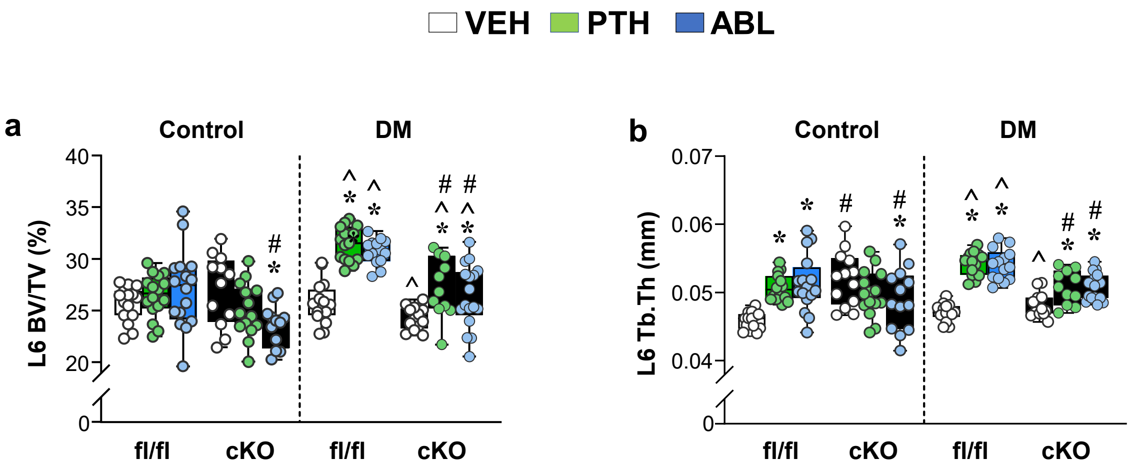

Supplementary Figure 5

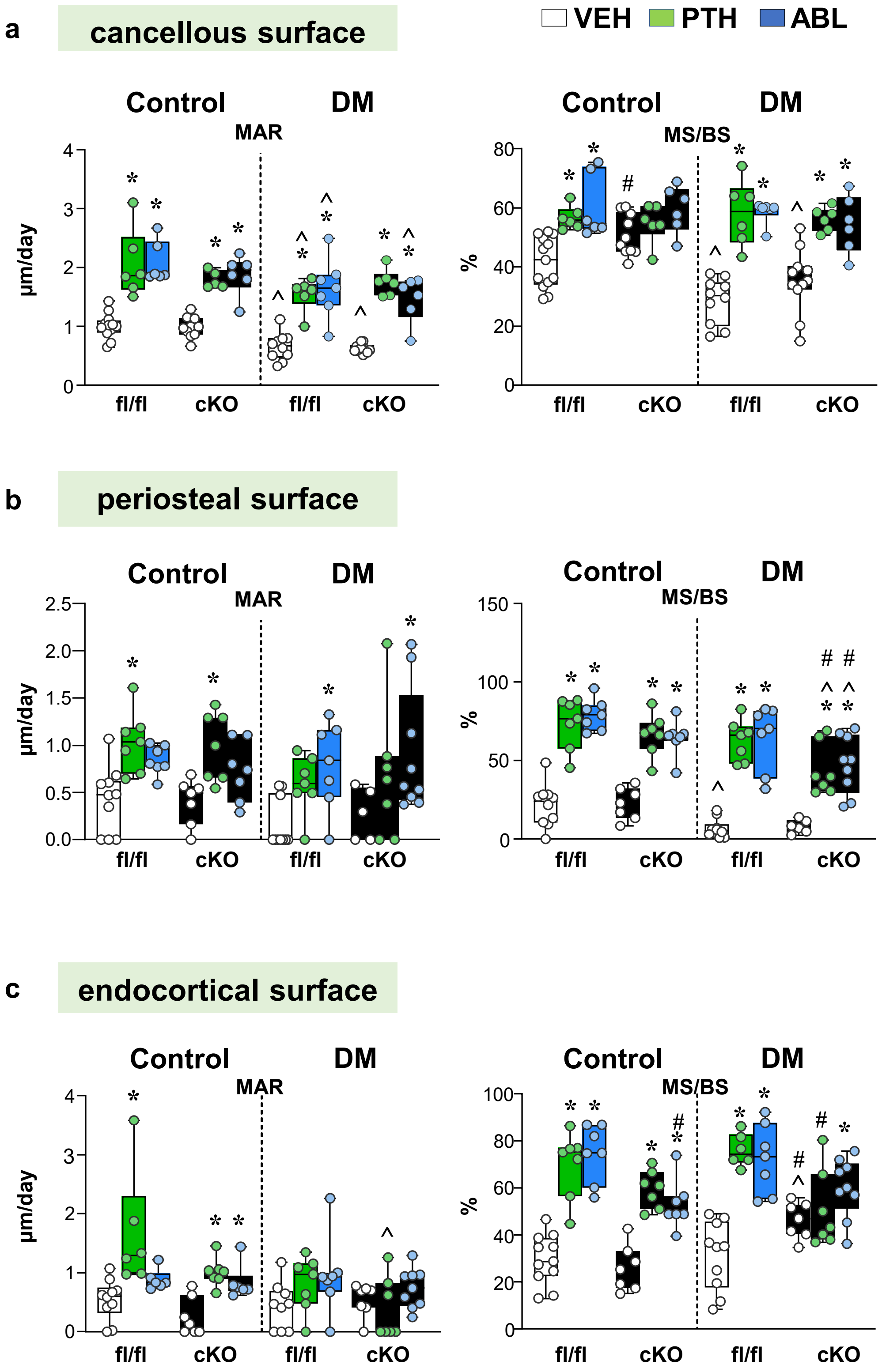

Supplement: Supplementary file 1 — Supplementary Figures [file 41413_2026_555_MOESM1_ESM.pdf]
