# Supplementary material for: DMP1-Cre expressing cells mediate the gain in bone mass and strength, but not the increase in bone remodeling, induced by ligands of the parathyroid hormone receptor
Source: Bone Res. 2026 Jul 30;14:77. doi: 10.1038/s41413-026-00555-z (PMC13424361; doi:10.1038/s41413-026-00555-z)
Supplement: Supplementary file 2 — Supplementary Figure Legends [file 41413_2026_555_MOESM2_ESM.docx]

**Supplementary Figure legends**

**Supplementary Figure 1: cKO mice exhibit higher total and spinal BMD compared to fl/fl littermates.** Longitudinal analysis of total (**a**), femoral (**b**) spinal (**c**) BMD, blood glucose concentration (**d**) and body weight (**e**), in control and DM fl/fl and cKO. n= 39-49/group at t0, n= 37-48/group at t1; n=39-48/group at t2; n=39-48/group at t3; and n= 12-17/group at t4 (vehicle only). # p<0.05 fl/fl versus fl/fl mice by Repeated Measures models.

**Supplementary Figure 2: Changes in fat and lean body mass induced by DM and treatments.** Longitudinal and final (t4) analysis of fat (**a, b**) and lean (**c, d**) body mass. For (**a, c**), n= 40-48/group at t0 and t1; n= 39-47/group at t2; n=38-47/group at t3; and n= 12-17/group at t4 (vehicle only); ^p<0.05 versus control mice by Repeated Measures models. For (**b, d**) n= (10-17). ^ p<0.05 versus control mice, * p<0.05 versus vehicle-treated mice, and # p<0.05 versus respective fl/fl mice, by three-way ANOVA with factors of DM, genotype, and treatment. Scapular (**e**), inguinal (**f**), and gonadal (**g**) fat weight corrected per body weight. n=11-17 mice/group. ^ p<0.05 versus control mice, by three-way ANOVA with factors of DM, genotype, and treatment. Each dot represents a mouse.

**Supplementary Figure 3: PTH and ABL corrected DM-induced deterioration of bone architecture in fl/fl mice but not in cKO mice.** Micro-CT representative images of the effects of DM and PTH/ABL on cancellous (**a**) and cortical (**b**) femoral bone; (**c**) cortical thickness of the femoral mid-diaphysis of fl/fl and cKO, control and DM mice. n=11-16 mice/group. ^ p<0.05 versus control mice, * p<0.05 versus respective vehicle-treated mice, and # p<0.05 versus respective fl/fl mice, by three-way ANOVA with factors of DM, genotype, and treatment. Each dot represents a mouse.

**Supplementary Figure 4: PTH and ABL increased and/or corrected DM-induced deterioration of bone architecture in fl/fl mice, although only partially in cKO mice.** Quantification of spinal (L6) cancellous bone (**a,b**) of fl/fl and cKO, control and DM mice. n=11-17 mice/group. ^ p<0.05 versus control mice, * p<0.05 versus respective vehicle-treated mice, and # p<0.05 versus respective fl/fl mice, by three-way ANOVA with factors of DM, genotype, and treatment. Each dot represents a mouse.

**Supplementary Figure 5.** **PTH and ABL increased bone formation in control and diabetic fl/fl mice and cKO mice.** MAR and MS/BS quantified by dynamic bone histomorphometric analysis of cancellous bone of the distal femur (**a**), periosteal (**b**), and endocortical (**c**) bone surfaces of the femoral mid-diaphysis. n=5-10 mice/group. ^ p<0.05 versus control mice, * p<0.05 versus respective vehicle-treated mice, and # p<0.05 versus respective fl/fl mice, by three-way ANOVA with factors of DM, genotype, and treatment.
