# Supplementary material for: DMP1-Cre expressing cells mediate the gain in bone mass and strength, but not the increase in bone remodeling, induced by ligands of the parathyroid hormone receptor
Source: Bone Res. 2026 Jul 30;14:77. doi: 10.1038/s41413-026-00555-z (PMC13424361; doi:10.1038/s41413-026-00555-z)
Supplement: Supplementary file 3 — Supplementary Material List [file 41413_2026_555_MOESM3_ESM.docx]

**Supplementary Material**

**Raw data excel files for:**

1. **Figure 2d**
2. **Figure 2e**
3. **Figure 2f**
4. **Figure 1b and Supplementary Figure 1d**
5. **Figure 1c and Supplementary Figure 1e**
6. **Figure 2a and Supplementary Figure 1a**
7. **Figure 2b and Supplementary Figure 1b**
8. **Figure 2c and Supplementary Figure 1c**
